# Homologues of the RNA binding protein RsmA in *Pseudomonas syringae* pv. *tomato* DC3000 exhibit distinct binding affinities with non‐coding small RNAs and have distinct roles in virulence

**DOI:** 10.1111/mpp.12823

**Published:** 2019-06-20

**Authors:** Yixin Ge, Jae Hoon Lee, Jun Liu, Ho‐wen Yang, Yanli Tian, Baishi Hu, Youfu Zhao

**Affiliations:** ^1^ College of Plant Protection and Key Laboratory of Integrated Management of Crop Diseases and Pests Nanjing Agricultural University Nanjing 210095 P. R. China; ^2^ Department of Crop Sciences University of Illinois at Urbana‐Champaign Urbana IL 61801 USA

**Keywords:** CsrA, post‐transcriptional regulation, *Pseudomonas syringae*, RsmA, type III secretion, virulence

## Abstract

*Pseudomonas syringae* pv*. tomato* DC3000 (*Pst*DC3000) contains five RsmA protein homologues. In this study, four were functionally characterized, with a focus on RsmA2, RsmA3 and RsmA4. RNA electrophoretic mobility shift assays demonstrated that RsmA1 and RsmA4 exhibited similar low binding affinities to non‐coding small RNAs (ncsRNAs), whereas RsmA2 and RsmA3 exhibited similar, but much higher, binding affinities to ncsRNAs. Our results showed that both RsmA2 and RsmA3 were required for disease symptom development and bacterial growth *in planta* by significantly affecting virulence gene expression. All four RsmA proteins, especially RsmA2 and RsmA3, influenced γ‐amino butyric acid utilization and pyoverdine production to some degree, whereas RsmA2, RsmA3 and RsmA4 influenced protease activities. A single RsmA, RsmA3, played a dominant role in regulating motility. Furthermore, reverse transcription quantitative real‐time PCR and western blot results showed that RsmA proteins, especially RsmA2 and RsmA3, regulated target genes and possibly other RsmA proteins at both transcriptional and translational levels. These results indicate that RsmA proteins in *Pst*DC3000 exhibit distinct binding affinities to ncsRNAs and have distinct roles in virulence. Our results also suggest that RsmA proteins in *Pst*DC3000 interact with each other, where RsmA2 and RsmA3 play a major role in regulating various functions in a complex manner.

## Introduction

The Gac/Rsm signal transduction system has been elaborately studied in many bacterial species (Babitzke and Romeo, 2007; Lapouge *et al.*, 2008). It has been widely reported that the GacS/GacA two‐component system (TCS) regulates pleotropic phenotypes, including virulence, stress responses, biofilm formation, production of extracellular enzymes, secondary metabolites and quorum sensing (Heeb and Haas, 2001; Lapouge *et al.*, 2008). The GacA homologues specifically initialize the transcription of non‐coding regulatory small RNAs (ncsRNAs), such as *csrB* and *csrC* in *Escherichia coli* (Jonas and Melefors, 2009; Martínez *et al.*, 2014), and *rsmW*, *rsmY*, *rsmZ* and *rsmV* in *Pseudomonas aeruginosa* (Janssen *et al*., 2018). These ncsRNAs contain numerous GGA motifs and bind and sequester the function of the RNA binding protein CsrA (carbon storage regulator) or its homologues RsmA and RsmE (repressor of secondary metabolites) (Reimmann *et al.*, 2005; Vakulskas *et al.*, 2015). As important post‐transcriptional regulators, the RsmA/CsrA family proteins inhibit translation or stability of transcripts of target genes by binding specific GGA motifs within apical loops of the RNA secondary structures in the 5′ untranslated regions (UTR), one of which overlaps or is close to the Shine–Dalgarno (SD) sequence or ribosome binding sites (RBSs) of target mRNAs, thus blocking ribosome access (Blumer *et al.*, 1999; Vakulskas *et al.*, 2015). On the other hand, the RsmA/CsrA family proteins can also positively regulate the expression of target genes. CsrA protects *flhDC* mRNA by inhibiting the 5' end‐dependent RNase E cleavage pathway in *E. coli* (Yakhnin *et al.*, 2013). RsmA activates the expression of *hrpG* gene by directly binding to the 5' UTR and stabilizing its mRNA in *Xanthomonas citri* (Andrade *et al.*, 2014).

The CsrA protein was first reported in *E. coli* by affecting glycogen biosynthesis and gluconeogenesis (Romeo *et al.*, 1993). Later, CsrA was deemed to be a global regulator, which regulates multiple important pathways in many bacteria (Timmermans and Van Melderon 2010). RsmA and RsmE are highly conserved CsrA homologues and play a major role in regulation of virulence in diverse pathogenic bacteria. In *X. citri* subsp. *citri* and *X. campestris* pv. *campestris*, an *rsmA* mutant caused significantly reduced virulence in the host plant, and delayed or completely abolished hypersensitive response (HR) in the non‐host plant tobacco (Andrade *et al.*, 2014; Chao *et al.*, 2008). In *Erwinia amylovora*, a *csrA* mutant did not induce HR on tobacco or cause disease on immature pear fruits. It was compromised in motility and had reduced exopolysaccharide (EPS) amylovoran and expression of type III secretion system (T3SS) genes. In addition, CsrA in *E. amylovora* may indirectly affected uptakes of antibiotics through the Rcs system (Ancona *et al.*, 2016; Ge *et al.*, 2018; Lee *et al.*, 2018). In *Pectobacterium carotovorum*, overexpression of *rsmA* inhibited motility, biofilm formation, EPS and secondary metabolite, antibiotics and pigment productions (Mukherjee *et al.*, 1996). Absence of *rsmA* resulted in less tissue‐macerating (soft‐rotting) in plant hosts through affecting quorum sensing required for extracellular lytic enzymes (Chatterjee *et al.*, 1995, 2003; Cui *et al.*, 2001). RsmA influenced expression of adhesion synthesis operon and indirectly affected virulence of *Yersinia pseudotuberculosis* (Heroven *et al.*, 2008). Loss of *csrA* greatly down‐regulated SPI‐1 intestinal epithelial cell invasion and other virulence gene expression in *S. typhimurium* (Lawhon *et al.*, 2003). In *Pseudomonas protegens*, RsmA and RsmE controlled metabolism and antibiotic biosynthesis (Reimmann *et al.*, 2005; Wang *et al.*, 2017). In *Pseudomonas putida*, RsmA, RsmE and RsmI negatively affected c‐di‐GMP pools and biofilm formation (Huertas‐Rosales *et al.*, 2016, 2017).

*Pseudomonas syringae* pv. *tomato* DC3000 (*Pst*DC3000) causes bacterial speck disease on tomato and *Arabidopsis thaliana* by secreting effectors through the T3SS and producing the phytotoxin coronatine (Zhao *et al.*, 2003). Extracellular protease, pyoverdine siderophore and alginate EPS also contribute to its virulence (Swingle *et al.*, 2008; Vargas *et al.*, 2013). Earlier bioinformatic studies showed that *P. syringae* pv. *tomato* possesses seven small RNAs, i.e. *rsmX* homologues (*rsmX1‐5*) as well as *rsmY* and *rsmZ* (Moll *et al.*, 2010). However, the molecular mechanism and virulence‐related target genes of the CsrA/RsmA proteins in *Pst*DC3000 remain elusive. A recent study identified five alleles of CsrA (RsmA) proteins present in *Pst*DC3000 and characterized three single mutants (*csrA1* to *csrA3*) (Ferreiro *et al.*, 2018). They showed that CsrA2 (RsmA2) is the most conserved member among 250 prokaryotic genomes examined, and CsrA3 (RsmA3) is nearly identical to CsrA2, but is present only in the *Pseudomonas fluorescens* group (RsmE). They further reported that CsrA3 and CsrA2 were found to play roles in motility, syringafactin and alginate production, and promote growth *in planta,* but not symptom development (Ferreiro *et al.*, 2018). Here, we labelled these CsrA proteins in *Pst*DC3000 as RsmA proteins because deduced amino acids of RsmA proteins in *Pst*DC3000 shared relatively higher identities and similarities to RsmA proteins from other *Pseudomonas* strains than to CsrA proteins from *E. coli* and *Erwinia* species (Fig. S1; Table S1). In our study, our major goal was to determine the roles of the *rsmA* genes in virulence and other virulent‐related phenotypes. We also explored the interactions between different RsmA proteins in *Pst*DC3000 and determined the binding affinities of these RsmA proteins to ncsRNAs.

## Results

### *In vitro* characterization of the *rsmA* mutants

We characterized four *rsmA* genes in *Pst*DC3000 by generating four overexpression strains (DC3000(pRsmA1, pRsmA2, pRsmA3, pRsmA4)), four single mutants (*rsmA1*, *rsmA2, rsmA3, rsmA4*), three double mutants (*rsmA2/rsmA3*, *rsmA2/rsmA4*, *rsmA3/rsmA4*), one triple mutant (*rsmA2/rsmA3/rsmA4*) and one quadruple mutant (*rsmA1*/*rsmA2/rsmA3/rsmA4*). Our results using overexpression strains indicate that overexpression of RsmA2, RsmA3 and Rsm4 in *Pst*DC3000 resulted in reduced pyoverdine production (Fig. S2A) and protease activities (Fig. S2B). Overexpression of RsmA2 and RsmA3 led to decreased ability in utilizing γ‐amino butyric acid (GABA), whereas overexpression of RsmA1 and RsmA4 resulted in enhanced GABA utilization (Fig. S3A). On the other hand, motility was slightly decreased only in *Pst*DC3000 overexpressing RsmA3 (Fig. S3B). Overexpressing of CsrA from *E. amylovora* in *Pst*DC3000 led to similar phenotypic changes with those of overexpressing RsmA2 of *Pst*DC3000 (Figs S2 and S3). The deduced amino acid sequence of CsrA of *E. amylovora* shared relatively higher identities and similarities to that of the RsmA2 than to RsmA3 of *Pst*DC3000 (Fig. S1; Table S1). We also observed that mutation of the *rsmA1*gene alone did not affect GABA utilization, protease activity and pyoverdine production (except a minor increase in motility) (Figs S4 and S5), suggesting that RsmA1 plays a very minimal role, as previously reported (Ferreiro *et al.*, 2018). Based on these results, we mainly focused on RsmA2, RsmA3 and RsmA4 for the majority of our studies.

### RsmA2 and RsmA3 are required for virulence and bacterial growth *in planta*

**Figure 1:**
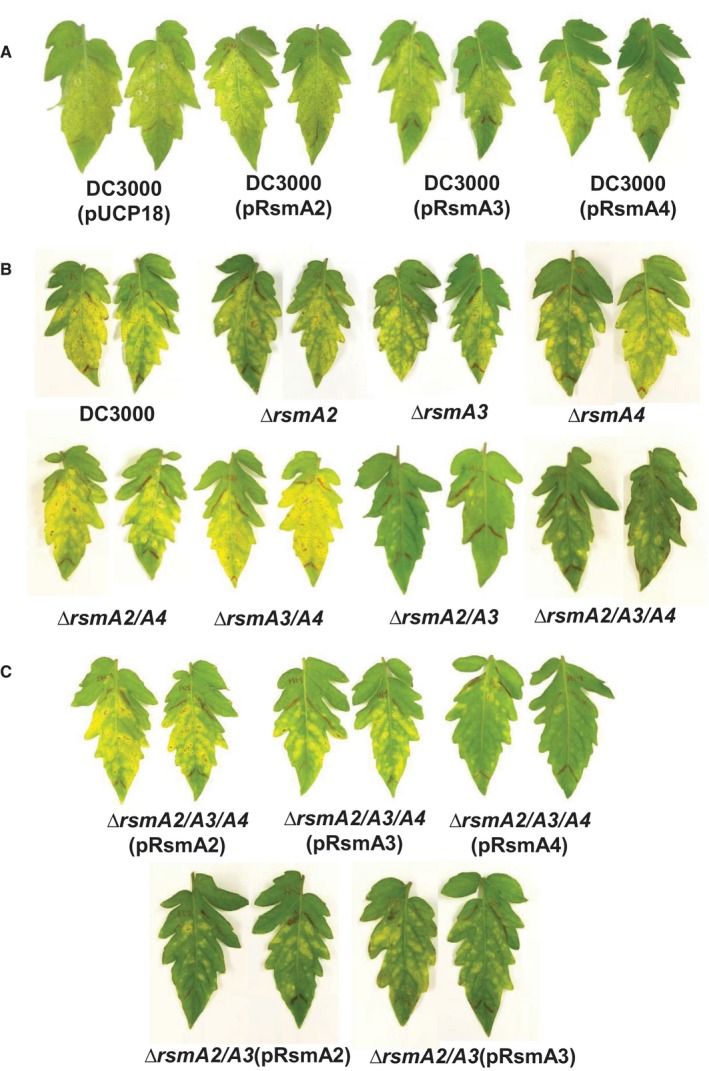
Virulence of *Pseudomonas syringae* pv. *tomato* DC3000, *rsmA* overexpression, *rsmA* mutants and complementation strains. (A) Disease symptoms caused by *Pst*DC3000(pUCP18), *Pst*DC3000(pRsmA2), *Pst*DC3000(pRsmA3) and *Pst*DC3000(pRsmA4) overexpression strains in tomato leaves. (B) Symptoms caused by *Pst*DC3000 and the *rsmA2*, *rsmA3*, *rsmA4*, *rsmA2/rsmA3*, *rsmA2/rsmA4*, *rsmA3/rsmA4* and *rsmA2/rsmA3/rsmA4* mutants in tomato leaves. (C) Symptoms caused by complementation strains of the *rsmA2/rsmA3* and the *rsmA2/rsmA3/rsmA4* mutants in tomato leaves. Pictures were taken at 7 days post‐inoculation. The experiment was repeated three times and similar results were obtained.

We inoculated plants using an infiltration method with a very low concentration of initial inoculum and found that *Pst*DC3000, all three overexpression strains, three single mutants (*rsmA2, rsmA3, rsmA4*) and two double mutants (*rsmA2/rsmA4* and *rsmA3/rsmA4*) exhibited similar disease symptoms (Fig. 1A,B). Interestingly, the *rsmA2/rsmA3* double mutant and the *rsmA2/rsmA3/rsmA4* triple mutant exhibited dramatically reduced symptoms, including both necrotic spots and the amount of chlorosis (Fig. 1B). Virulence of the *rsmA2/rsmA3/rsmA4* triple mutant could be restored by complementation with either the *rsmA2* or *rsmA3* gene, but not with the *rsmA4* gene (Fig. 1C). Virulence of the *rsmA2/rsmA3* double mutant could partially be recovered by either *rsmA2* or *rsmA3* (Fig. 1C). These findings suggest that both RsmA2 and RsmA3 are required for virulence.

**Figure 2:**
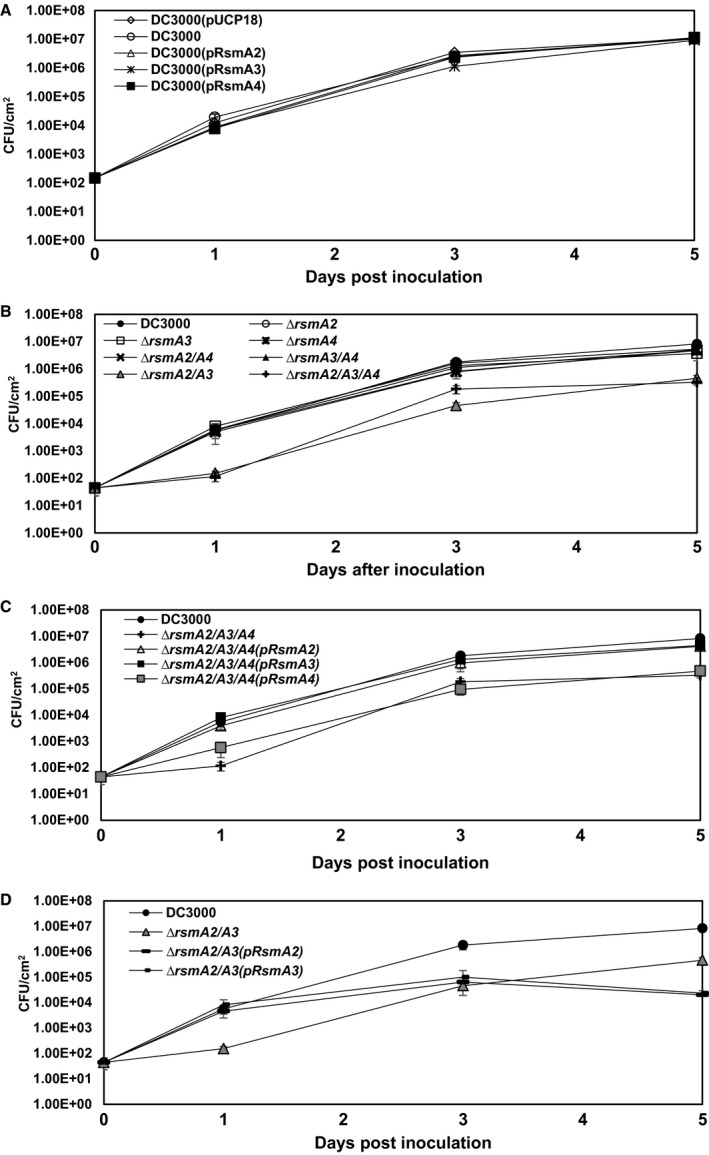
Bacterial growth of *Pseudomonas syringae* pv. *tomato* DC3000, *rsmA* overexpression, *rsmA* mutants and complementation strains in tomato. (A) *Pst*DC3000, *Pst*DC3000(pUCP18), *Pst*DC3000(pRsmA2), *Pst*DC3000(pRsmA3) and *Pst*DC3000(pRsmA4) overexpression strains. (B) *Pst*DC3000 and the *rsmA2*, *rsmA3*, *rsmA4*, *rsmA2/rsmA3*, *rsmA2/rsmA4*, *rsmA3/rsmA4* and *rsmA2/rsmA3/rsmA4* mutants. (C) *Pst*DC3000, the *rsmA2/rsmA3/rsmA4 *mutant and its complementation strains. (D) *Pst*DC3000, the *rsmA2/rsmA3* and its complementation strains. Bacterial growth was monitored at 0, 1, 3 and 5 days post‐inoculation. Vertical bars represent standard deviations. The experiment was repeated three times and similar results were obtained.

We also monitored bacterial growth *in planta* at 0, 1, 3 and 5 days post‐inoculation (dpi). No significant difference was found among *Pst*DC3000, three overexpressed strains and three single mutants as well as the *rsmA2/rsmA4* and the *rsmA3/rsmA4* double mutants (Fig. 2A,B). Bacterial growth for the *rsmA2/rsmA3* double mutant and the *rsmA2/rsmA3/rsmA4* triple mutant was about 5–10‐fold lower than those of all other strains (Fig. 2B), and bacterial growth could be completely rescued in the triple mutant by expression of either the *rsmA2* or *rsmA3* gene, but not the *rsmA4* gene (Fig. 2C). Expression of the *rsmA2* or *rsmA3* gene in the *rsmA2/rsmA3* double mutant could partially restore bacterial growth in tomato leaves (Fig. 2D). In order to rule out the possibility that the defect of bacterial growth *in planta* was due to defects in their abilities to grow *in vitro*, we determined bacterial growth in KB medium. Though some mutants showed delay in growth, all the mutants reached a similar level to the wild‐type after 24 h of growth (Fig. S6). All the overexpression strains and mutants could still elicit HR on non‐host tobacco leaves (Fig. S7). Overall, these results support the finding that RsmA2 and RsmA3 play an important role in the interaction of *Pst*DC3000 with tomato plants, and suggest that these proteins might have functional redundancy.

### Expression of T3SS, coronatine and alginate genes in *Pst*DC3000 is regulated by RsmA2, RsmA3 and RsmA4 to varying degrees

**Figure 3:**
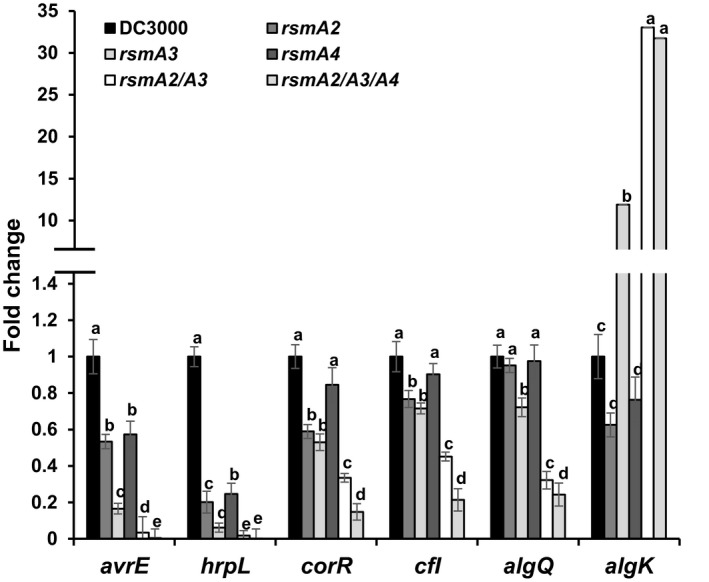
Expression of selected virulence genes of *Pseudomonas syringae* pv. *tomato* DC3000 and the *rsmA* mutants as compared to the wild type. Expression of *avrE*, *hrpL*, *corR*, *cfl*, *algQ* and *algK* genes in the *rsmA2*, *rsmA3*, *rsmA4*, *rsmA2/rsmA3* and *rsmA2/rsmA3/rsmA4* mutant strains as compared to that of *Pst*DC3000 grown in a *hrp*‐inducing minimal medium at 6 h determined by qRT‐PCR. The *rpoD* gene was used as a control. Vertical bars represent the standard deviations of mean ratio. Bars marked with the same letter are not significantly different (*P* < 0.05). The experiment was repeated three times, and three technical replicates were included for each of the two biological samples per experiment.

In order to determine how mutation of the *rsmA* genes affects virulence, expression of selected virulence‐related genes in five mutants was quantified. First, expression of *avrE* and *hrpL* in all five mutants was reduced (Fig. 3), especially in the *rsmA2/rsmA3* double mutant and the *rsmA2/rsmA3/rsmA4* triple mutant, where expression of *avrE* and *hrpL* was more than 30–50‐ and 250–1000‐fold lower than that of the wild type (WT), respectively (Fig. 3). In the single mutant, the effect of the *rsmA3* gene on expression of *hrpL* and *avrE* genes was much stronger than that of the *rsmA2* and *rsmA4* genes. These results suggest that RsmA2, RsmA3 and RsmA4 synergistically influence the expression of T3SS genes in *Pst*DC3000, and further indicate that RsmA3 plays a major role.

Expression of coronatine‐related genes, i.e. the *corR* and *cfl* genes, was also reduced in four of the five mutants. Expression of the *corR* and *cfl* genes in the *rsmA2/rsmA3* double mutant was decreased about 3‐ and 2.2‐fold than that of the wild type, respectively, while in the *rsmA2/rsmA3/rsmA4* triple mutant, they were also down‐regulated about 6.7‐ and 4.8‐fold, respectively (Fig. 3). Both *corR* and *cfl* gene expression was slightly down‐regulated in the *rsmA2* and *rsmA3* mutants, but not in the *rsmA4* mutant (Fig. 3). These results suggest that RsmA2 and RsmA3 synergistically regulate coronatine gene expression in *Pst*DC3000, whereas RsmA4 might also have a minor role when both RsmA2 and RsmA3 are absent.

Expression pattern for the *algQ* gene was reduced in the *rsmA3*, *rsmA2/rsmA3* and *rsmA2/rsmA3/rsmA4* mutants (Fig. 3). However, expression of *algK* gene was increased in all three mutants lacking the *rsmA3* gene. Expression of *algK* was up‐regulated about 11‐fold in the *rsmA3* mutant and more than 30‐fold in both the *rsmA2/rsmA3* double mutant and the *rsmA2/rsmA3/rsmA4* triple mutant than that in the WT  (Fig. 3). These results suggest that different regulation mechanisms exist for the *algQ* and *algK* genes in *Pst*DC3000 by the RsmA proteins.

### Protease activities are influenced by RsmA2, RsmA3 and RsmA4

**Figure 4:**
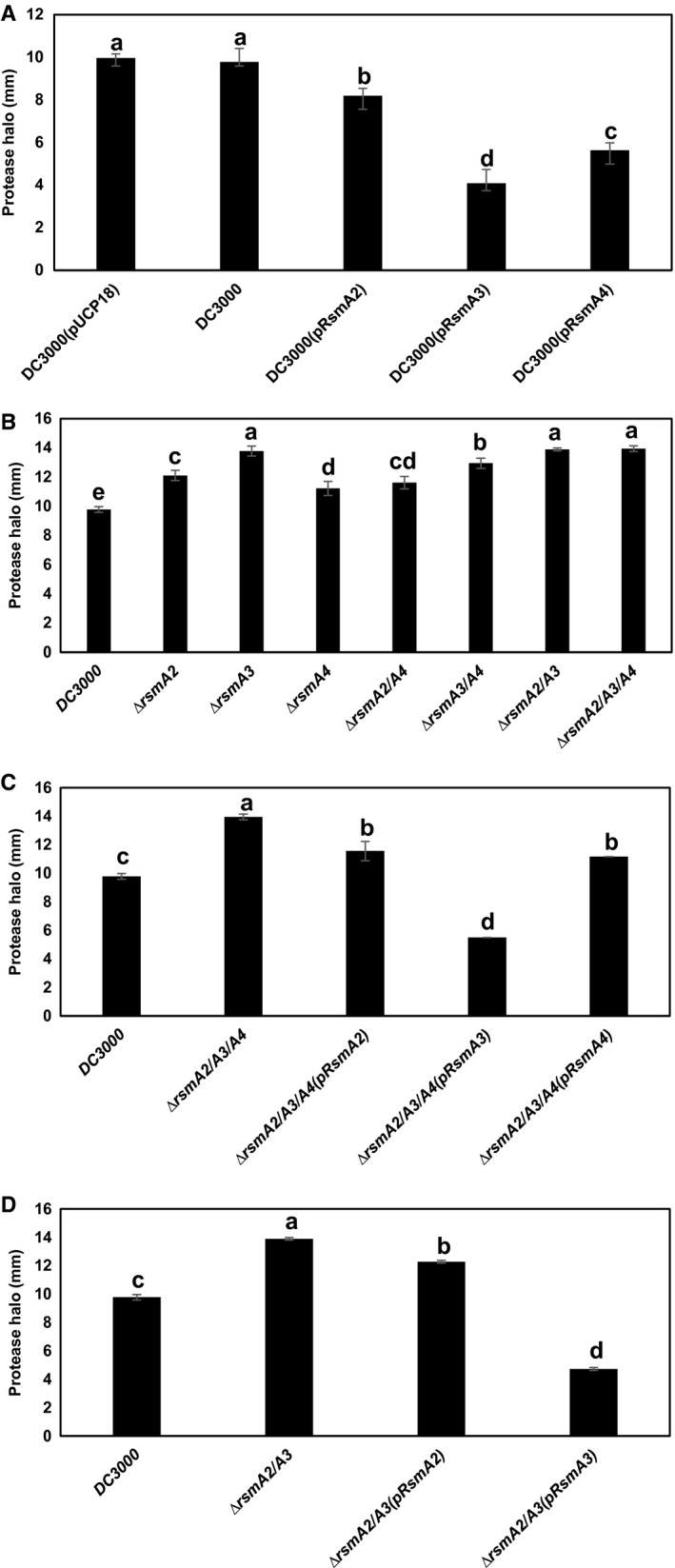
Diameter of halo zones of protease activities. (A) *Pst*DC3000, *Pst*DC3000(pUCP18), *Pst*DC3000(pRsmA2), *Pst*DC3000(pRsmA3) and *Pst*DC3000(pRsmA4) overexpression strains. (B) *Pst*DC3000 and the *rsmA2*, *rsmA3*, *rsmA4*, *rsmA2/rsmA3*, *rsmA2/rsmA4*, *rsmA3/rsmA4* and *rsmA2/rsmA3/rsmA4* mutants. (C) *Pst*DC3000, the *rsmA2/rsmA3/rsmA4* mutant and its complementation strains. (D) *Pst*DC3000, the *rsmA2/rsmA3* mutant and its complementation strains. All strains were grown on NYG agar plates containing 0.75% skimmed milk at room temperature. Diameters were measured after 24 h of incubation. Vertical bars represent standard deviations. Bars marked with the same letter are not significantly different (*P *< 0.05). The experiment was repeated three times with three replicate and similar results were obtained.

Overexpression of RsmA in *Pst*DC3000 exhibited reduced protease activities compared to that of *Pst*DC3000 (Figs 4A and S8A). In contrast, the *rsmA2*, *rsmA3* and *rsmA4* deletion mutants all exhibited increased protease activities compared to *Pst*DC3000 (Figs 4B and S8B). The protease activities of the *rsmA2/rsmA4* and *rsmA3/rsmA4* mutants were also slightly increased (Figs 4B and S8B), whereas the protease activities for the *rsmA2/rsmA3*, *rsmA2/rsmA3/rsmA4* and *rsmA1/rsmA2/rsmA3/rsmA4* mutants were similar to each other and increased to the level of the *rsmA3* single mutant (Figs 4B, S4B and S8B). Complementation of the *rsmA2/rsmA3/rsmA4* and* rsmA2/rsmA3* mutant with the *rsmA2* gene partially restored protease activities, whereas complementation with the *rsmA3* gene led to reduced protease activities (Figs 4CD and S8C). In conclusion, these results indicate that RsmA3 plays a major role in regulating protease activity, whereas Rsm2 and RsmA4 also negatively influence protease activity in *Pst*DC3000.

### The effect of RsmA proteins on pyoverdine production

**Figure 5:**
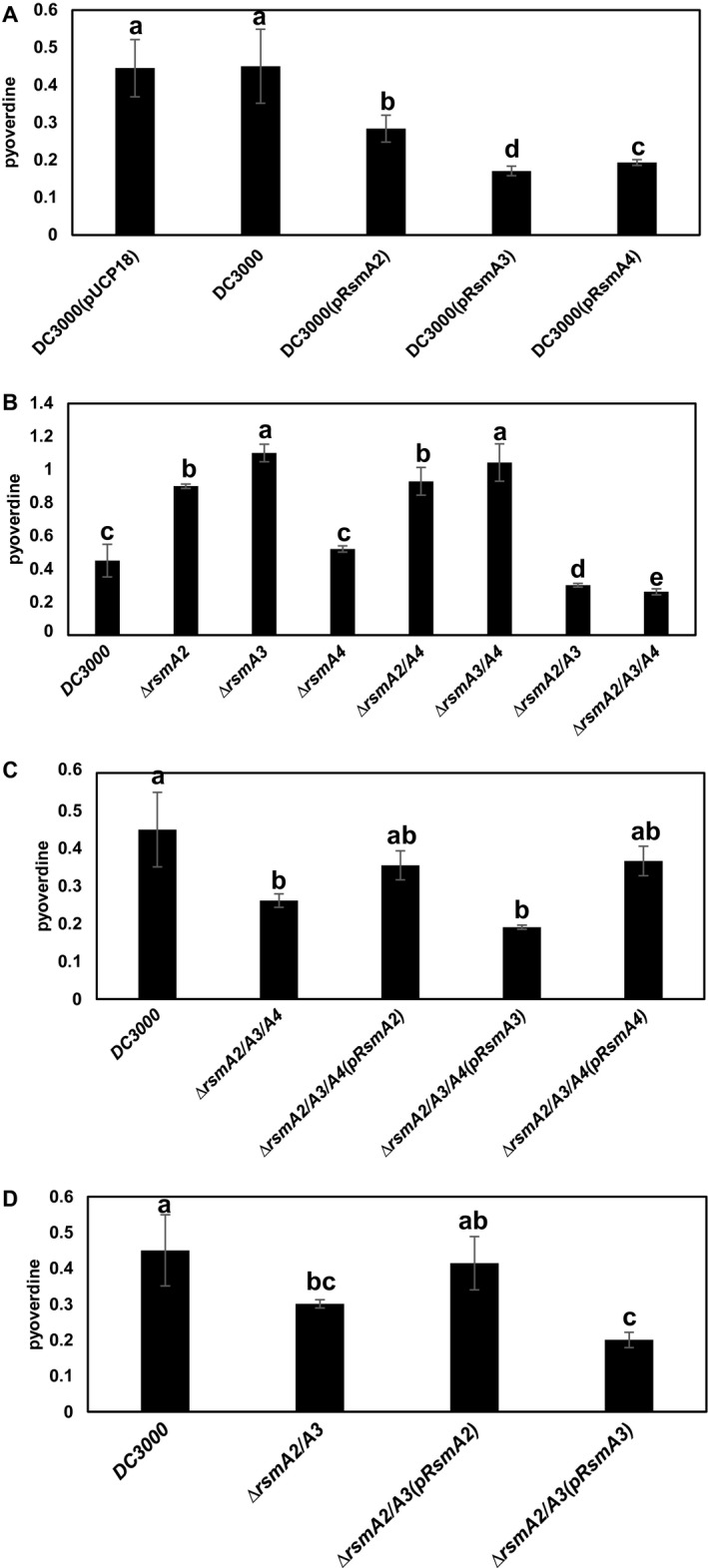
Quantification of pyoverdine production. (A) *Pst*DC3000, *Pst*DC3000(pUCP18), *Pst*DC3000(pRsmA2), *Pst*DC3000(pRsmA3) and *Pst*DC3000(pRsmA4) overexpression strains. (B) *Pst*DC3000 and the *rsmA2*, *rsmA3*, *rsmA4*, *rsmA2/rsmA3*, *rsmA2/rsmA4*, *rsmA3/rsmA4* and *rsmA2/rsmA3/rsmA4* mutants. (C) *Pst*DC3000, the *rsmA2/rsmA3/rsmA4* mutant and its complementation strains. (D) *Pst*DC3000, the *rsmA2/rsmA3* mutant and its complementation strains. Pyoverdine production was quantified by measuring the absorbance at OD_405_ of culture supernatants diluted 2:1 in 100 mM Tris‐HCl (pH 8.0) and normalized at OD_600_ of bacterial suspensions. Data were presented as relative fluorescence levels (A_405_/A_600_). All strains were grown in MG medium at 28 °C for 24 h. Vertical bars represent standard deviations. Bars marked with the same letter are not significantly different (*P* < 0.05). The experiment was repeated three times with three replicates and similar results were obtained.

One characteristic of fluorescent pseudomonads is the production of pyoverdine as a siderophore and virulence‐related signal molecule (Imperi *et al.*, 2009). Similar to the protease activity results, overexpression of *rsmA2*, *rsmA3* or *rsmA4* led to varying reduced pyoverdine production, where overexpression of the *rsmA3* gene exhibited the strongest negative effect (Figs 5A and S9A). In contrast, deletion of the *rsmA2* and *rsmA3* genes, but not the *rsmA4* or *rsmA1 *genes, led to increased pyoverdine production (Figs 5B, S4A and S9A). Furthermore, pyoverdine production in the *rsmA2/rsmA4* and *rsmA3/rsmA4* double mutants was similar to that in the *rsmA2* and *rsmA3* single mutants, respectively (Figs 5B and S9B). When both *rsmA2* and *rsmA3* were deleted, pyoverdine production in the *rsmA2/rsmA3* double mutant and the *rsmA2/rsmA3/rsmA4* triple mutants was significantly lower compared to DC3000, but similar to that of the *rsmA3* overexpression strains (*P* < 0.05, Figs 5A,B and S9A,B). However, the *rsmA1/rsmA2/rsmA3/rsmA4* quadruple mutant exhibited increased pyoverdine production compared to the *rsmA2/rsmA3* double and *rsmA2/rsmA3/rsmA4* triple mutants (Fig. S4A). Complementation of the *rsmA2/rsmA3/rsmA4* mutant with either *rsmA2* or *rsmA4*, but not the *rsmA3* gene, partially restored pyoverdine production to the wild‐type level (Figs 5C and S9C). Similarly, complementation of the *rsmA2/rsmA3* mutant by expressing the *rsmA2*, but not the *rsmA3* gene, partially recovered its pyoverdine production (Figs 5D and S9C). These results indicate that all four RsmA proteins influenced pyoverdine production in *Pst*DC3000 to some degree. These results also suggest that expression levels of RsmA2 and RsmA3, especially RsmA3, might be important in pyoverdine production, and RsmA1 might also play a role in pyoverdine production when RsmA2, RsmA3 and RsmA4 are all deleted.

### RsmA3 negatively regulates motility in *Pst*DC3000

In contrast to protease activity and pyoverdine production, motility was only slightly decreased in *Pst*DC3000 overexpressing the *rsmA3* gene and slightly increased in the single *rsmA1* and *rsmA3* mutants and the *rsmA3/rsmA4* double mutant (Figs S3B, S5B, S10A,B and S11A,B). Similar to pyoverdine production, when both *rsmA2* and *rsmA3* were deleted, motility of the *rsmA2/rsmA3* double mutant, the *rsmA2/rsmA3/rsmA4* triple mutant and the *rsmA1*/*rsmA2/rsmA3/rsmA4* quadruple mutant was significantly reduced as compared to other strains (*P* < 0.05, Figs S5B, S10B and S11B). Motility can be rescued for the *rsmA2/rsmA3* double mutant and the *rsmA2/rsmA3/rsmA4* triple mutant by the *rsmA2* or *rsmA4* gene, but not the *rsmA3* gene as described above for pyoverdine production (Figs S10C and S11CD). These results indicate that although RsmA1 may also suppress motility, RsmA3 plays a dominant role in regulating motility in *Pst*DC3000, and also suggest that RsmA2 could affect motility when interacting with RsmA3.

### The effect of RsmA on GABA utilization

**Figure 6:**
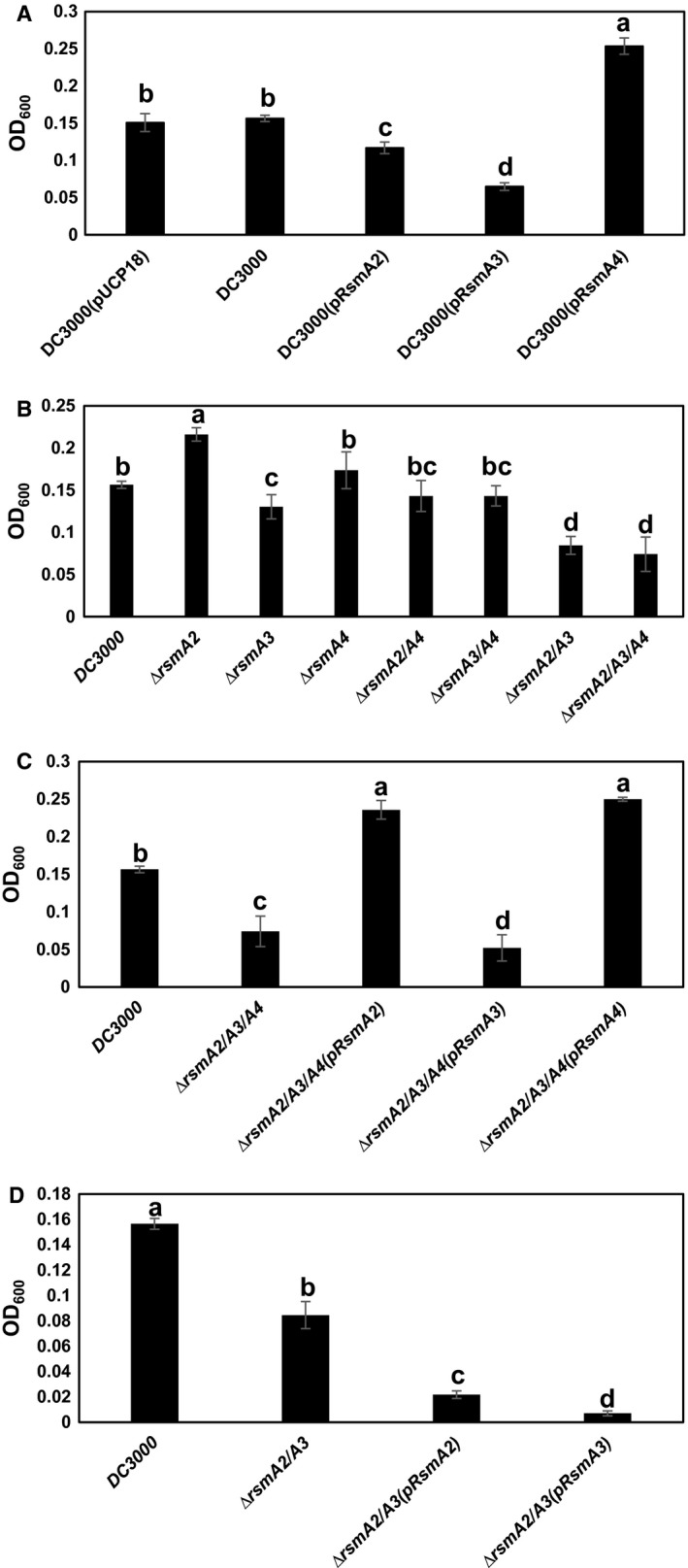
Growth of *Pseudomonas syringae* pv. *tomato* DC3000, *rsmA* overexpression, *rsmA* mutants and complementation strains in GABA. (A) *Pst*DC3000, *Pst*DC3000(pUCP18), *Pst*DC3000(pRsmA2), *Pst*DC3000(pRsmA3) and *Pst*DC3000(pRsmA4) overexpression strains. (B) *Pst*DC3000 and the *rsmA2*, *rsmA3*, *rsmA4*, *rsmA2/rsmA3*, *rsmA2/rsmA4*, *rsmA3/rsmA4* and *rsmA2/rsmA3/rsmA4* mutants. (C) *Pst*DC3000, the *rsmA2/rsmA3/rsmA4* mutant and its complementation strains. (D) *Pst*DC3000, the *rsmA2/rsmA3* mutant and its complementation strains. All the strains were grown in modified MG medium (replacing mannitol and l‐glutamic acid in MG medium with 10 mM γ‐amino butyric acid) at 28 °C and bacterial growth was monitored by measuring OD_600_ at 24 h. Vertical bars represent standard deviations. Bars marked with the same letter were not significantly different (*P* < 0.05). The experiment was repeated three times with three replicates and similar results were obtained.

Non‐protein amino acid GABA is highly abundant in tomato apoplast and *Pst*DC3000 can utilize GABA as a sole carbon and nitrogen source, thus utilization of GABA might affect its survival in planta (Rico and Preston, 2008). Overexpression of the *rsmA2* and *rsmA3* genes in *Pst*DC3000 led to decreased ability in utilizing GABA, whereas overexpression of the *rsmA4* gene resulted in enhanced GABA utilization (Fig. 6A). In contrast, deletion of the *rsmA2* and *rsmA3* genes increased or decreased GABA utilization, respectively, whereas no effect was found in the *rsmA1* and *rsmA4* single mutants or in the *rsmA2/rsmA4* and *rsmA3/rsmA4* double mutants and the *rsmA1*/*rsmA2/rsmA3/rsmA4* quadruple mutant (Figs 6B and S5A). Similar to motility and pyoverdine production, when both the *rsmA2* and *rsmA3* genes were deleted, GABA utilization in the *rsmA2/rsmA3* double mutant and the *rsmA2/rsmA3/rsmA4* triple mutant was significantly decreased compared to other strains, but was similar to that of the *rsmA3* overexpression strain (*P* < 0.05, Fig. 6A,B). Complementation of the *rsmA2/rsmA3/rsmA4* mutant with either the *rsmA2* or *rsmA4* gene increased GABA utilization to the same level as the *rsmA4* overexpression strain (Fig. 6A,C), whereas complementation with the *rsmA3* gene led to GABA utilization as low as that of the *rsmA3* overexpression strain (Fig. 6A,C). Surprisingly, when the *rsmA2/rsmA3* double mutant complemented with either the *rsmA2* or *rsmA3* gene, it resulted in significantly decreased ability in utilizing GABA (*P* < 0.05, Fig. 6D). These results indicate that all four RsmA proteins influence GABA utilization to varying degrees in *Pst*DC3000. Furthermore, when RsmA2, RsmA3 and RsmA4 were all absent, RsmA1 could also influence GABA utilization, suggesting that the interaction between these four RsmA proteins in influencing GABA utilization is very complicated.

### Expression of the *rsmA* genes in *Pst*DC3000

**Figure 7:**
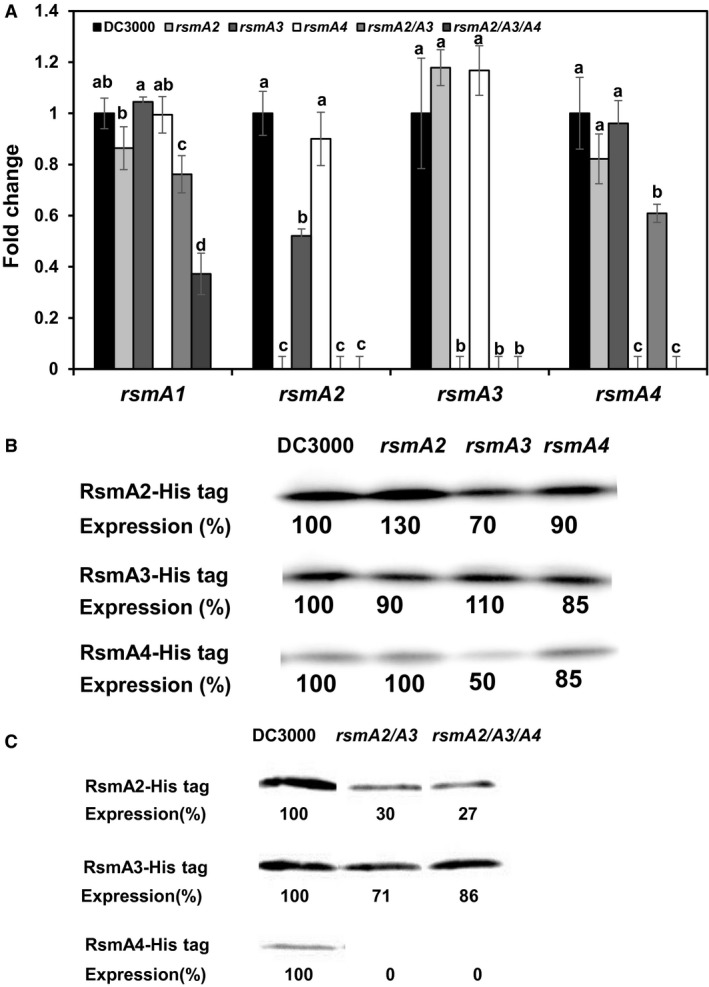
Expression of the *rsmA *genes and abundance of the RsmA2, RsmA3 and RsmA4 proteins in *Pseudomonas syringae* pv. *tomato* DC3000 and the *rsmA* mutants. (A) Expression of the *rsmA1*, *rsmA2*, *rsmA3* and *rsmA4* genes in the *rsmA2*, *rsmA3*, *rsmA4*, *rsmA2/rsmA3* and *rsmA2/rsmA3/rsmA4* mutant strains as compared to that of *Pst*DC3000 grown in a *hrp*‐inducing minimal medium at 6 h and determined by qRT‐PCR. The *rpoD* gene was used as a control. Vertical bars represent the standard deviations of mean ratio. Bars marked with the same letter are not significantly different (*P* < 0.05). (B) Abundance of RsmA2‐His6, RsmA3‐His6 and RsmA4‐His6 in the *rsmA2*, *rsmA3* and *rsmA4* mutant strains as compared to that of *Pst*DC3000 grown in HMM medium for 24 h at 18 °C. (C) Abundance of RsmA2‐His6, RsmA3‐His6 and RsmA4‐His6 in the *rsmA2*/*rsmA3* and *rsmA2*/*rsmA3/rsmA4* mutant strains as compared to that of *Pst*DC3000 grown in HMM medium for 24 h at 18 °C.

In order to give a glimpse of the complex interaction among RsmA proteins in *Pst*DC3000, we first determined the expression of the *rsmA* genes using reverse transcription quantitative real‐time PCR (qRT‐PCR) in HMM medium. Expression of *rsmA2* was decreased about 2‐fold in the *rsmA3* single mutant compared to that of the wild‐type and *rsmA4* mutant (Fig. 7A). No significant change was observed for the *rsmA3* gene in the mutant strains tested. However, in the *rsmA2*/*rsmA3* double mutant, expression of the *rsmA4* and *rsmA1 *genes was slightly down‐regulated, whereas expression of the *rsmA1* gene was significantly down‐regulated in the *rsmA2*/*rsmA3*/*rsmA4* triple mutant (*P* < 0.05, Fig. 7A). These results suggest that RsmA3 positively regulates *rsmA2* expression. Furthermore, the results also suggest that RsmA2 and RsmA3 might synergistically activate the expression of the *rsmA4* gene, whereas RsmA2, RsmA3 and RsmA4 synergistically promote the expression of the *rsmA1* gene.

Western blot analyses showed that the abundance of the RsmA2 and RsmA4 proteins was decreased about 30% and 50% in the *rsmA3* single mutant strain, respectively, whereas the abundance of RsmA3 protein remained mostly unchanged in the *rsmA2* and *rsmA4* single mutants (Figs 7B and S12A). Furthermore, the abundance of the RsmA2 protein was significantly decreased (70% less) in the *rsmA2/rsmA3* double mutant and the *rsmA2/rsmA3/rsmA4* triple mutant as compared to the WT, whereas the abundance of the RsmA4 protein was not detectable in the *rsmA2/rsmA3* double mutant and the *rsmA2/rsmA3/rsmA4* triple mutant (Figs 7C and S12B). Interestingly, the abundance of the RsmA3 protein was also decreased about 30% in the *rsmA2/rsmA3* double mutant, but only slightly decreased in the *rsmA2/rsmA3/rsmA4* triple mutant (Figs 7C and S12B). These results suggest that RsmA3 influences the expression of RsmA2 and RsmA4 proteins. These results also suggest that RsmA2 might self‐regulate itself and reciprocally influence RsmA3 expression, whereas RsmA2 and RsmA3 together might also affect the expression of RsmA4 proteins.

### RsmA proteins have distinct binding affinities to ncsRNAs

**Figure 8:**
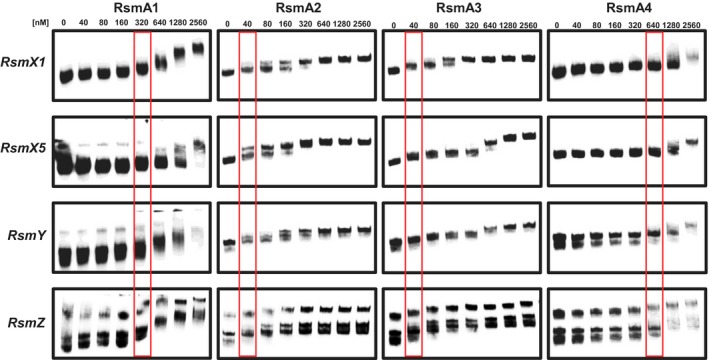
RsmA proteins exhibit distinct binding affinities to ncsRNAs. To compare RNA‐binding affinity to different ncsRNAs (*RsmX1*, *RsmX5*, *RsmY* and *RsmZ*), four RsmA proteins (RsmA1, RsmA2, RsmA3 and RsmA4) were purified and subjected to RNA gel shift assays. Different concentrations of four proteins (nM) are indicated above each lane. The red frame shows the minimal concentration of proteins where band shift could be observed.

To compare RNA‐binding affinities to different ncsRNAs, four RsmA proteins (RsmA1, RsmA2, RsmA3 and RsmA4) were purified and subject to RNA gel shift assays (Fig. 8). Since previous sequence analysis of five *rsmX* ncsRNAs revealed the same secondary structure with five GGA motifs in the hairpin loop (Moll *et al.*, 2010), only *rsmX1* and *rsmX5* as well as *rsmY* and *rsmZ* were selected for the analysis. In all four ncsRNAs tested, a band shift was observed at 40 nM for RsmA2 and RsmA3, at 320 nM for RsmA1, and at 640 nM for RsmA4. These results indicate that all ncsRNAs of *Pst*DC3000 exhibit similar binding affinity to different RsmA homologues, while RsmA homologues have distinct binding affinities to ncsRNAs in the following order from strongest to weakest: RsmA2 = RsmA3 > RsmA1 > RsmA4.

## Discussion

Since 1993, the CsrA/RsmA homologues have been extensively studied in human and plant pathogens as well as plant‐associated microorganisms. In a previous report, three paralogues, i.e. *csrA1*, *csrA2* and *csrA3* in *Pst*DC3000, were evaluated for their roles in motility, alginate biosynthesis, syringafactin production and virulence, and the authors concluded that CsrA1 to CsrA3 were not required for virulence (Ferreiro *et al.*, 2018). However, in our study we demonstrated that RsmA2 and RsmA3 were required for virulence in *Pst*DC3000 and bacterial growth *in planta*, and RsmA4 might also play a minor role in virulence. We also provided evidence that RsmA2 and RsmA3 regulate genes at both transcriptional and post‐transcriptional levels and interactions between themselves are very complicated. Moreover, we showed that RsmA proteins in *Pst*DC3000 exhibited distinct binding affinities to ncsRNAs, which might explain the distinguishing role each RsmA protein plays in affecting various phenotypes.

RsmA (CsrA) plays a critical role in virulence among many pathogenic bacteria (Ancona *et al.*, 2016; Andrade *et al.*, 2014; Barnard *et al.*, 2004). It has been reported that deletion of a single *csrA* gene in *Pst*DC3000 did not affect virulence, but bacterial growth was reduced in the *csr2* and *csr3* mutants (Ferreiro *et al.*, 2018). In our study, we confirmed that deletion of a single *rsmA* gene did not change its ability to cause disease. However, we found that deletion of both *rsmA2* and *rsmA3* significantly affects disease symptom development and bacterial growth, suggesting that both RsmA2 and RsmA3 are required for *Pst*DC3000 virulence and bacterial growth *in planta*. These results also suggest that RsmA proteins in *Pst*DC3000 exhibit function redundancies in controlling virulence factors.

The T3SS and phytotoxin coronatine are major pathogenicity and virulence factors in *Pst*DC3000, respectively (Zhao *et al.*, 2003). The ability of *Pst*DC3000 to multiply in plant tissue and promote symptom development is dependent on the translocation of many effector proteins to target specific host proteins and interfere with plant innate immune signalling systems (Feng and Zhou, 2012; Mudgett, 2005). The non‐host specific toxin coronatine increases disease severity by suppressing stomata closure and promoting the jasmonic acid (JA) signalling pathway to suppress salicylic acid (SA)‐mediated defence responses (Brooks *et al.*, 2004; Elizabeth and Bender, 2007; Uppalapati *et al.*, 2007; Zhao *et al.*, 2003). Our results show that transcription levels of *avrE*, *hrpL*, *corR* and *cfl* are positively regulated by RsmA proteins at various degrees, indicating that RsmA proteins synergistically regulate virulence factors and contribute to virulence, and further suggesting that RsmA proteins in *Pst*DC3000 have function redundancy. We also demonstrated that RsmA2 and RsmA3 play a major role, whereas RsmA1 and RsmA4 play a minor role in virulence.

In addition, our results show that RsmA3 negatively regulates *algK* gene, which is responsible for alginate synthesis, whereas RsmA2 positively affect *algK* expression. However, expression of *algK* increased drastically in mutants deleting both RsmA2 and RsmA3. Our results are consistent with previous reports that *algD* is up‐regulated in both the *rsmA* mutant of *P. aeruginosa* and the *csrA3* mutant of *Pst*DC3000 (Burrowes *et al*., 2006; Ferreiro *et al.*, 2018). In contrast, expression of *algQ*, encoding a global regulatory protein of alginate biosynthesis (Ambrosi *et al.*, 2005; Kim *et al.*, 1998; Schlictman *et al.*, 1995), was significantly decreased when both *rsmA2* and *rsmA3* were deleted, indicating that RsmA3 and RsmA2 synergistically regulate AlgQ at transcriptional level. It is possible that AlgK is negatively regulated by AlgQ, which needs to be further verified.

The potential interplay between different Rsm proteins has been investigated in some *Pseudomonas* strains (Kay *et al.*, 2005; Morris *et al.*, 2013; Zha *et al.*, 2014). In *P. fluorescens*, RsmE expression was negatively regulated by RsmA and RsmE, which regulate itself (Reimmann *et al.*, 2005). Both RsmA and RsmE, which are closely related to RsmA2 and RsmA3, respectively, negatively affect their own expression in *P. putida* (Huertas‐Rosales *et al.*, 2016). Furthermore, RsmA and RsmF translation is repressed by specific binding of RsmA to *rsmA* and *rsmF* mRNA *in vitro* at post‐transcriptional level in *P. aeruginosa* (Marden *et al.*, 2013). In our study, one of the novel findings is that RsmA3 positively regulates RsmA2 at transcriptional level. This result might explain why many phenotypes were most significant when both RsmA2 and RsmA3 were absent. In other words, RsmA3 probably is on the top of the RsmA regulatory cascade in *Pst*DC3000. Interestingly, our results suggest that expression of the *rsmA1* gene, which is present in most *Pseudomonas* strains, might be suppressed by the synergistic action of RsmA2, RsmA3 and RsmA4 at transcriptional level. In addition, we demonstrated that in the *rsmA3* mutant, the abundance of the RsmA2 and RsmA4 protein was significantly decreased, indicating that RsmA3 positively affects RsmA2 and RsmA4 at post‐transcriptional and translational levels. Furthermore, the abundances of the RsmA2, RsmA3 and RsmA4 proteins were all decreased in *rsmA2* and *rsmA3* double mutants as compared to the wild‐type and the *rsmA3* single mutant, further suggesting that RsmA2 and RsmA3 might synergistically and reciprocally influence the expression of RsmA2, RsmA3 and RsmA4 proteins at the post‐transcriptional level. Future studies should focus on illustrating the exact interaction among these RsmA proteins in *Pst*DC3000.

It is assumed that RsmA/CsrA proteins bind to target mRNAs at the conserved GGA motif located in the loops of the hairpin structure within the 5ʹ UTR, whereas ncsRNAs, containing numerous GGA motifs, sequester their functions (Vakulskas *et al.*, 2015). We demonstrated that RsmA protein homologues have distinct binding affinities to ncsRNAs, whereas RsmA2 and RsmA3 in *Pst*DC3000 exhibited similar binding affinities, which are much stronger than those of RsmA1 and RsmA4. These results are consistent with previous findings that RsmA and RsmE in *P. fluorescens* have similar binding affinities to ncsRNAs (Kay *et al.*, 2005; Reimmann *et al*., 2005). These results also provide evidence as why RsmA2 and RsmA3 are more important than RsmA1/RsmA4 in regulating various phenotypes. The question that remains unanswered is, since the major residues in binding to GGA motif are well conserved among these RsmA proteins, why do they display distinct binding affinities to ncsRNAs?

In summary, the RsmA/CsrA family protein has long been deemed an important and pleiotropic post‐transcriptional regulator in many bacteria and is extensively involved in the gene regulatory network (Vakulskas *et al.*, 2015). In our current study, we demonstrated that RsmA proteins in *Pst*DC3000 modulate virulence and bacterial growth *in planta*, and regulate protease activity, pyoverdine production, utilization of GABA and motility. We also demonstrated that RsmA proteins in *Pst*DC3000 exhibit distinct binding affinities to fine‐tune the expression of target genes, both negatively and positively. We further provided evidence for the existence of regulatory interactions between different RsmA proteins both at transcriptional and translational levels; however, the exact regulatory mechanism remains unknown. In the future, it is worth exploring potential direct or indirect regulation pathways as well as interaction among the RsmA proteins. Furthermore, identifying direct targets of RsmA proteins in the regulation of virulence factors should be a priority.

## Experimental Procedures

### Bacterial strains, plasmids and culture conditions

The bacterial strains and plasmids used in this study are listed in Table 1. *Pseudomonas syringae* pv. *tomato* strains were cultured on King’s medium B (KB). For T3SS gene expression, an *hrp*‐inducing minimal medium (HMM), supplemented with 10 mM fructose as carbon source, was used (Chatnaparat *et al.*, 2015). Luria‐Bertani (LB) broth was utilized for routine growth of *E. coli* strain at 37 °C. Antibiotics were used at the following concentrations when appropriate: 100 μg/mL rifampicin, 50 μg/mL kanamycin, 100 μg/mL ampicillin, 15 μg/mL tetracycline and 100 μg/mL spectinomycin. All the primers used are listed in Supplementary Table S2.

**Table 1:** Bacterial strains and plasmids used in this study.

Strains, plasmids	Description	Reference or source
***Pseudomonas syringae* pv*. tomato***		
DC3000	Wild type, Rif^r ^spontaneous resistance	
Δ*rsmA1(Pspto1629*)	Km^r^ *, rsmA1*::Kan*,* DC3000 derivative	This study
Δ*rsmA2*(*Pspto1844)*	Km^r^ *, rsmA2*::Kan*,* DC3000 derivative	This study
Δ*rsmA3(Pspto3566)*	Km^r^ *, rsmA3*::Kan*,* DC3000 derivative	This study
Δ*rsmA4(Pspto3943)*	Km^r^ *, rsmA4*::Kan*,* DC3000 derivative	This study
Δ*rsmA2/rsmA4*	Km^r^ *, rsmA2* and *rsmA4*::Kan*,* DC3000 derivative	This study
Δ*rsmA3/rsmA4*	Km^r^ *, rsmA3* and *rsmA4*::Kan*,* DC3000 derivative	This study
Δ*rsmA2/rsmA3*	Km^r^ *, rsmA3* and *rsmA2*::Kan*,* DC3000 derivative	This study
Δ*rsmA2/rsmA3/rsmA4*	Km^r^ *, rsmA3*, *rsmA4* and *rsmA2*::Kan*,* DC3000 derivative	This study
Δ*rsmA1/rsmA2/rsmA3/rsmA4*	Km^r^ *, rsmA3*, *rsmA4*, *rsmA2* and *rsmA1*::Kan*,* DC3000 derivative	This study
***E. amylovora***		
Ea1189	Wild type, isolated from apple	Wang *et al.*, 2010
***E. coli***		
DH10B	*F − mcrA Δ(mrr‐hsdRMS‐mcrBC) Φ80lacZΔM15 ΔlacX74 recA1 endA1 araΔ139 Δ(ara, leu)7697 galU galK λ—rpsL (StrR) nupG*	Invitrogen (Carlsbad, CA, USA)
BL21(DE3)	F^‐^ompT hsdS_B_(rB^‐^mB^‐^) gal dcm (DE3)	Invitrogen (Carlsbad, CA, USA)
**Plasmids**		
pUCP18	*E. coli*‐*Pseudomonas* shuttle vector, Ap^r^	Norrander *et al.*, 1983
pTok2	ColE1 replicon, suicide plasmid, Tc^r^	Kitten and Willis, 1996
pKD13	FRT‐Kan‐FRT, oriR6K, Ap^r^, Km^r^	Datsenko and Wanner, 2000
pFLP2‐omega	Suicide vector encoding flp recombinase, *sacB*, Sp^r^	Chatnaparat *et al.*, 2015
pGEM‐T Easy	PCR cloning vector, Ap^r^	Promega
pET‐42b	*E. coli* His‐tag expression vector, Amp^r^	Novagen
pTok2‐*rsmA1del*	*rsmA1*::Kan from overlapping PCR cloned into pTok2, Tc^r^, Km^r^	This study
pTok2‐*rsmA2del*	*rsmA2*::Kan from overlapping PCR cloned into pTok2, Tc^r^, Km^r^	This study
pTok2‐*rsmA3del*	*rsmA3*::Kan from overlapping PCR cloned into pTok2, Tc^r^, Km^r^	This study
pTok2‐*rsmA4del*	*rsmA4*::Kan from overlapping PCR cloned into pTok2, Tc^r^, Km^r^	This study
pRsmA1	995‐bp fragment containing *rsmA1 *gene with native promoter cloned into pUCP18, Ap^r^	This study
pRsmA2	989‐bp fragment containing *rsmA2 *gene with native promoter cloned into pUCP18, Ap^r^	This study
pRsmA3	989‐bp fragment containing *rsmA3 *gene with native promoter cloned into pUCP18, Ap^r^	This study
pRsmA4	995‐bp fragment containing *rsmA4 *gene with native promoter cloned into pUCP18, Ap^r^	This study
pCsrA	894‐bp fragment containing *csrA* gene with native promoter from *E. amylovora* Ea1189 cloned into pUCP18, Ap^r^	This study
pRsmA2‐His6	827‐bp fragment containing *rsmA2 *gene with native promoter and C‐terminal His‐tag in pUCP18, Ap^r^	This study
pRsmA3‐His6	827‐bp fragment containing *rsmA3 *gene with native promoter and C‐terminal His‐tag in pUCP18, Ap^r^	This study
pRsmA4‐His6	833‐bp fragment containing *rsmA4 *gene with native promoter and C‐terminal His‐tag in pUCP18, Ap^r^	This study
pRsmX1	120‐bp DNA fragment carrying the *rsmX1* gene in pGEM‐T easy, Ap^r^	This study
pRsmX5	112‐bp DNA fragment carrying the *rsmX5* gene in pGEM‐T easy, Ap^r^	This study
pRsmY	126‐bp DNA fragment carrying the *rsmY* gene in pGEM‐T easy, Ap^r^	This study
pRsmZ	132‐bp DNA fragment carrying the *rsmZ* gene in pGEM‐T easy, Ap^r^	This study
pET42b‐RsmA1	213‐bp fragment containing *rsmA1 *gene and C‐terminal His‐tag in pET‐42b, Ap^r^	This study
pET42b‐RsmA2	207‐bp fragment containing *rsmA2 *gene and C‐terminal His‐tag in pET‐42b, Ap^r^	This study
pET42b‐RsmA3	207‐bp fragment containing *rsmA3 *gene and C‐terminal His‐tag in pET‐42b, Ap^r^	This study
pET42b‐RsmA4	213‐bp fragment containing *rsmA4 *gene and C‐terminal His‐tag in pET‐42b, Ap^r^	This study

Rif^r^, Km^r^, Tc^r^, Ap^r^ and Sp^r^ indicate rifampicin, kanamycin, tetracycline, ampicillin and spectinomycin resistance, respectively.

### Construction of deletion mutants

Deletion mutations of *rsmA1*, *rsmA2*, *rsmA3*, *rsmA4*, *rsmA2/rsmA3*, *rsmA2/rsmA4*, *rsmA3/rsmA4*, *rsmA2/rsmA3/rsmA4* and *rsmA1*/*rsmA2/rsmA3/rsmA4* were generated using splice overlap extension mutagenesis as described previously (Chatnaparat *et al.*, 2015). Briefly, in a first round of PCRs, about 1 kb upstream and downstream fragments of *rsmA1*, *rsmA2*, *rsmA3* and *rsmA4* in *Pst*DC3000 were amplified using primers unique to these regions (Table S2). The two PCR products contained ends overlapping with sequences at both ends of the kanamycin resistance cassette (Datsenko and Wanner, 2000). The FRT‐Km‐FRT forward and reverse primers were used amplified FRT‐flanked kanamycin cassette. In a subsequent overlap extension PCR, these three fragments were amplified into a single fragment. The final fragment was cloned into the pTok2 suicide vector digested with *Sma*I, resulting in pTok2‐*rsmA1del*, pTok2‐*rsmA2del*, pTok2‐*rsmA3del* and pTok2‐*rsmA4del*, respectively. To generate marker‐less mutants, plasmid pFLP2‐omega expressing FLP recombinase was introduced into the mutant and transformants were plated on KB plates containing spectinomycin, resulting in the loss of the kanamycin resistance cassette. To generate double mutants, plasmid pTok2‐*rsmA4del* or pTok2‐*rsmA2del* was transferred into the *rsmA2* and *rsmA3* marker‐less mutants. For generating the *rsmA2/rsmA3/rsmA4* triple and *rsmA1*/*rsmA2/rsmA3/rsmA4* quadruple mutants, the plasmids pTok2‐*rsmA2del* and pTok2‐*rsmA1del* were transferred into the *rsmA3/rsmA4* and *rsmA2/rsmA3/rsmA4* marker‐less mutant strains, respectively.

### Complementation of mutants and generation of overexpression strains

For complementation of the *rsmA* mutants, a 1 kb fragment containing the native promoter and the *rsmA* genes was amplified by PCR and cloned into the pUCP18 vector to yield plasmids pRsmA1, pRsmA2, pRsmA3 and pRsmA4. The resulting plasmids were sequenced at the University of Illinois at Urbana‐Champaign core sequencing facility. The final plasmids were introduced into the corresponding marker‐less deletion mutants and *Pst*DC3000 by electroporation. In addition, the promoter and the *csrA* gene from *E. amylovora* Ea1189 strain were also amplified and cloned into the pUCP18 vector and introduced into *Pst*DC3000.

### Motility, pyoverdine production, protease activity and GABA utilization assays

For motility assay, cells were grown overnight in KB medium, harvested and washed in phosphate‐buffered saline (PBS). Cells were resuspended in PBS and 2 μL of bacterial suspensions (OD_600_ = 2.0) were spotted onto the centre of motility plates (0.3% KB agar). The plates were incubated for 48 h at room temperature and the motility of bacterial cells was visually examined at 24 and 48 h post‐inoculation. The experiments were performed three times, with three biological replicates per treatment.

Pyoverdine product was detected in mannitol–glutamate (MG) medium as previously described (Ambrosi *et al.*, 2005; Chatnaparat *et al.*, 2015; Imperi *et al.*, 2009; Park *et al.*, 2010). Bacterial cells of overnight cultures in KB were washed in PBS, resuspended to an OD_600 _of 0.05 in MG medium and incubated with shaking at 28 °C for 24 h. Pyoverdine was quantified by measuring the absorbance at 405 nm of culture supernatants diluted 2:1 in 100 mM Tris‐HCl (pH 8.0) and normalized with OD_600_. To visualize pyoverdine product, bacterial cells were resuspended in PBS to a final concentration of OD_600_ = 0.3, and 2 μL bacterial suspensions were spotted onto MG agar plates. Plates were incubated at 28 °C and observed under UV light after 48 h. The experiments were performed three times, with three biological replicates per treatment.

For protease activity, all strains were grown in KB overnight, rinsed and resuspended in PBS to a density of OD_600_ = 1. Bacterial suspensions (2 μL) were applied onto NYG (peptone yeast glycerol medium; 5 g/L peptone, 3 g/L yeast extract, 20 g/L glycerol) agar plates containing 0.75% skimmed milk. Plates were incubated at room temperature for 3 days prior to examination and measurement of the diameter of halo zones. The experiments were repeated three times, with three biological replicates each.

For GABA utilization assays, bacterial cells of overnight cultures in KB were washed and resuspended to OD_600_ = 0.02 in modified MG medium by replacing mannitol and l‐glutamic acid in MG medium with 10 mM GABA (Chatnaparat *et al.*, 2015). Cells were grown overnight at 28 °C, and bacterial growth was monitored by measuring OD_600_. The experiments were repeated three times, with three biological replicates each.

### Virulence assay and bacterial growth in tomato

Tomato, *Solanum lycopersicum* ‘Big Daddy Hybrid’ plants were grown in a greenhouse. About 3–4 weeks after transplanting, leaves were infiltrated with bacterial suspension at about 5 × 10^4^ CFU/mL (diluted from original suspension at OD_600_ = 0.1) using a needleless syringe. Bacteria were recovered from plants by taking three samples from three leaves at the site of infiltration using a disk punch (three disks per strain) at 0, 1, 3, 5 days post‐inoculation (dpi). Leaf disks were homogenized by mechanical disruption using pestles in PBS. Serial 10‐time dilutions of the tissue homogenates were plated on LB plates, and the number of CFUs per disk (cm^2^) was calculated. For virulence assay, disease symptoms were recorded at 7 dpi. The experiment was repeated three times.

### RNA isolation and reverse transcription quantitative real‐time PCR

After 6 h incubation in HMM at 18 °C, 4 mL of RNA protect reagent (Qiagen, Hilden, Germany) was added to 2 mL of bacterial culture mixed by vortex and incubated at room temperature for 5 min. Cells were harvested by centrifugation and RNA was extracted using RNeasy® mini kit (Qiagen, Hilden, Germany) according to the manufacturer’s instructions. DNase I treatment was performed with TURBO DNA‐free kit (Ambion, TX, USA) and RNA was quantified using Nano‐drop ND100 spectrophotometer (Nano‐Drop Technologies, Wilmington, DE, USA). One microgram of total RNA was reverse transcribed using Superscript III reverse transcriptase (Invitrogen, Carlsbad, CA, USA) following the manufacturer’s instructions. One microgram of cDNA was used as the template for reverse transcription quantitative real‐time PCR (qRT‐PCR). PowerUp SYBR^®^ Green PCR master mix (Applied Biosystems, CA, USA) was used to detect gene expression of selected genes. qRT‐PCR amplifications were performed using the StepOnePlus Real‐Time PCR system (Applied Biosystems, CA, USA) under the following conditions: 50 °C for 2 min and 95 °C for 2 min followed by 40 cycles of 95 °C for 15 s and 60 °C for 1 min. The dissociation curve was measured after the programme was completed and gene expression was analysed with the relative quantification (ΔΔC_t_) method using the *rpoD* gene as an endogenous control. The experiment was repeated three times, and three technical replicates were included for each of the two biological samples per experiment.

### Western blot

The DNA fragments containing the native promoters and coding sequences of the *rsmA2*, *rsmA3* and *rsmA4* genes with a 6‐His tag at the C‐terminus were cloned into pUCP18. The resulting plasmids were transformed by electroporation into the *Pst*DC3000 and mutants. For western blot, equal amounts of bacterial cells grown in HMM containing 10 mM fructose at 18 °C for 24 h were collected. Cell lysates were resolved by sodium dodecyl sulphate polyacrylamide gel electrophoresis (SDS‐PAGE) and transferred to polyvinylidene fluoride membrane (Millipore, Billerica, MA, USA). After blocking with 5% milk in PBS, membranes were probed with 1.0 μg/mL rabbit anti‐His antibodies (GenScript, Piscataway, NJ, USA), followed by horseradish peroxidase‐linked anti‐rabbit IgG antibodies (Amersham Bioscience, Uppsala, Sweden) diluted 1:10 000. Immunoblots were developed using enhanced chemiluminescence reagents (Pierce, Rockford, IL, USA) and visualized using an ImageQuant LAS 4010 CCD camera (GE Healthcare, South Plainfield, NJ, USA). As a loading control, a duplicate protein gel was incubated in staining solution with shaking overnight and then incubated in destaining solution with shaking until the bands could be observed clearly. The experiment was performed at least two times.

### Protein expression and purification

The *rsmA1*, *rsmA2*, *rsmA3* and *rsmA4* genes were amplified by PCR (Table S2). The PCR products were cloned into pET‐42b vector with a C‐terminal hexahistidine tag to construct the plasmids pET42b‐RsmA1, pET42b‐RsmA2, pET42b‐RsmA3 and pET42b‐RsmA4. The plasmids were electroporated into *E. coli* BL21 (DE3), and the transformants were induced with 0.5 mM isopropyl β‐d‐1‐thiogalactopyranoside (IPTG) for 3 h at 37 °C to obtain the recombinant protein. Cells were centrifuged and lysed by centrifuging at 8635***g*** for 45 min at 4 °C. The supernatant was passed through an Ni‐NTA affinity chromatographic column to obtain the proteins (GE Healthcare, Uppsala, Sweden).

### RNA electrophoretic mobility shift assays

Full‐length sequences of each ncsRNA (*rsmX1*, *rsmX5*, *rsmY* and *rsmZ*) were PCR‐amplified and cloned into the pGEM‐T Easy vector (Promega, Madison, WI, USA). RNA probes were prepared from the cloned vector as a template using MEGAshortscript kit (Thermo Fisher Scientific, Waltham, MA, USA) and labelled with biotin using Pierce RNA 3ʹ end biotinylation kit (Thermo Fisher Scientific), according to the manufacturer’s instruction. RNA gel shift assays were performed using Lightshift® chemiluminescent RNA EMSA kit (Thermo Fisher Scientific) with slight modifications. Briefly, reaction mixtures were prepared in volumes of 10 μL, containing 2 nM of biotin‐labelled target RNA, 1 × binding buffer, 5% glycerol and 0.4 units RNase inhibitor. After 20 min incubation with different amounts of proteins at room temperature, 5 × loading buffer was added to the binding reaction. Protein–RNA complexes were separated on a 6% native polyacrylamide gel in 0.5 × TBE buffer (44.5 mM Tris‐base, 44.5 mM boric acid and 1 mM EDTA) and UV‐light crosslinked to a nylon membrane. Chemiluminescence was visualized using an ImageQuant LAS 4010 CCD camera (GE Healthcare, Piscataway, NJ, USA).

### Statistical analysis

Statistical comparison among different strains or conditions was performed by one‐way ANOVA and the Student–Newman–Keuls test (*P* = 0.05) to analyse the data.

## Competing Interests

The authors have declared that no competing interests exist.

## Supporting information


**Fig. S1** (A) Phylogenetic tree of RsmA/CsrA proteins from *E. coli*, *S. enterica*, *E. amylovora* and *Pseudomonas* strains. (B) Alignment of deduced amino acids of different RsmA/CsrA proteins in *E. amylovora* and three *Pseudomonas* strains. The deduced amino acid sequences of RsmA/CsrA proteins were aligned and analysed by GeneDoc software (Nicholas *et al*., 1997). Phylogenetic tree of RsmA/CsrA proteins was made by MEGA5 (Tamura *et al*., 2001). The GenBank accession numbers are *E. coli *CsrA: BAA16558; *S. enterica* subsp. *enterica* CsrA: NP_461747; *E. amylovora* CFBP1430 CsrA: CBA19758; Host factor‐I: CBA23141; *P. aeruginosa* PAO1 RsmA: AAG04294; *P. aeruginosa* UCBPP‐PA14 RsmN: BAK92751; *P. fluorescens* F113 RsmA: ABW16952; RsmE: ABW16953; *P. fluorescens* A506 RsmE: AFJ58988; *P. syringae *pv. *syringae* B728a RsmA1: YP_236820; RsmA2: YP_236624; RsmA3: YP_236409; *P. syringae* pv. *tomato* DC3000 RsmA1: AAO55149; RsmA2: AAO55363; RsmA3: AAO57040; RsmA4: AAO57404; RsmA5: YP_003355050.

**Fig. S2** Effect of RsmA1 of *P. syringae *pv*. tomato* DC3000 and CsrA of *E.* *amylovora *on pyoverdine production and protease activity. (A) Pyoverdine production by *Pst*DC3000(pRsmA1) and *Pst*DC3000(pCsrA) overexpression strains and compared with *Pst*DC3000(pUCP18), *Pst*DC3000(pRsmA2), *Pst*DC3000(pRsmA3) and *Pst*DC3000(pRsmA4) overexpression strains. (B) Protease activity by *Pst*DC3000(pRsmA1) and DC3000(pCsrA) overexpression strains and compared with *Pst*DC3000(pUCP18), *Pst*DC3000(pRsmA2), *Pst*DC3000(pRsmA3) and *Pst*DC3000(pRsmA4) overexpression strains. Vertical bars represent standard deviations. One‐way ANOVA and the Student–Newman–Keuls test (*P* = 0.05) were used to analyse the data. Bars marked with the same letter are not significantly different (*P* < 0.05). The experiment was repeated three times and similar results were obtained.

**Fig. S3** Effect of RsmA1 of *P. syringae *pv*. tomato* DC3000 and CsrA of *E. amylovora *on GABA utilization and swimming motility. (A) Growth of *Pst*DC3000(pRsmA1) and *Pst*DC3000(pCsrA) overexpression strains as compared with *Pst*DC3000(pUCP18), *Pst*DC3000(pRsmA2), *Pst*DC3000(pRsmA3) and *Pst*DC3000(pRsmA4) overexpression strains in GABA. All the strains were grown in modified MG medium (replacing mannitol and l‐glutamic acid in MG medium with 10 mM GABA) at 28 °C and bacterial growth was monitored by measuring OD_600_ at 24 h. Vertical bars represent standard deviations. Bars marked with the same letter are not significantly different (*P* < 0.05). The experiment was repeated three times and similar results were obtained. (B) Motility of *Pst*DC3000(pRsmA1) and *Pst*DC3000(pCsrA) overexpression strains compared with *Pst*DC3000(pUCP18), *Pst*DC3000(pRsmA2), *Pst*DC3000(pRsmA3) and *Pst*DC3000(pRsmA4) overexpression strains. Vertical bars represent standard deviations. One‐way ANOVA and the Student–Newman–Keuls test (*P* = 0.05) were used to analyse the data. Bars marked with the same letter were not significantly different (*P* < 0.05). The experiment was repeated three times and similar results were obtained.

**Fig. S4** Effect of the *rsmA1* mutant and the *rsmA1*/*rsmA2*/*rsmA3*/*rsmA4* quadruple mutant of *P. syringae *pv*. tomato* DC3000 on pyoverdine production and protease activity. (A) Pyoverdine production by the *rsmA1* mutant and the *rsmA1*/*rsmA2*/*rsmA3*/*rsmA4* quadruple mutant and compared with *Pst*DC3000, *Pst*DC3000(pRsmA1), the *rsmA2*/*rsmA3* and the *rsmA2*/*rsmA3*/*rsmA4* mutant strains. (B) Protease activity by the *rsmA1* mutant and the *rsmA1*/*rsmA2*/*rsmA3*/*rsmA4* quadruple mutant and compared with *Pst*DC3000, *Pst*DC3000(pRsmA1) and the *rsmA2*/*rsmA3* and *rsmA2*/*rsmA3*/*rsmA4* mutant strains. Vertical bars represent standard deviations. One‐way ANOVA and the Student–Newman–Keuls test (*P* = 0.05) were used to analyse the data. Bars marked with the same letter are not significantly different (*P* < 0.05). The experiment was repeated three times and similar results were obtained.

**Fig. S5** Effect of the *rsmA1* mutant and the *rsmA1*/*rsmA2*/*rsmA3*/*rsmA4* quadruple mutant of *P. syringae *pv*. tomato* DC3000 on GABA utilization and motility. (A) Growth of the *rsmA1* mutant and the *rsmA1*/*rsmA2*/*rsmA3*/*rsmA4* quadruple mutant as compared with *Pst*DC3000, *Pst*DC3000(pRsmA1) and the *rsmA2*/*rsmA3* and *rsmA2*/*rsmA3*/*rsmA4* mutant strains. All the strains were grown in modified MG medium (replacing mannitol and l‐glutamic acid in MG medium with 10 mM GABA) at 28 °C and bacterial growth was monitored by measuring OD_600_ at 24 h. Vertical bars represent standard deviations. Bars marked with the same letter are not significantly different (*P* < 0.05). The experiment was repeated three times and similar results were obtained. (B) Motility of the *rsmA1* mutant and the *rsmA1*/*rsmA2*/*rsmA3*/*rsmA4* quadruple mutant as compared with *Pst*DC3000, *Pst*DC3000(pRsmA1) and the *rsmA2*/*rsmA3* and *rsmA2*/*rsmA3*/*rsmA4* mutant strains. Vertical bars represent standard deviations. One‐way ANOVA and the Student–Newman–Keuls test (*P* = 0.05) were used to analyse the data. Bars marked with the same letter are not significantly different (*P* < 0.05). The experiment was repeated three times and similar results were obtained.

**Fig. S6** Growth of *P. syringae* pv. *tomato* DC3000, *rsmA* overexpression and mutant strains. (A) *Pst*DC3000, *Pst*DC3000(pUCP18), *Pst*DC3000(pRsmA1), *Pst*DC3000(pRsmA2), *Pst*DC3000(pRsmA3), *Pst*DC3000(pRsmA4) and *Pst*DC3000(pCsrA) overexpression strains. (B) *Pst*DC3000 and the *rsmA2*, *rsmA3* and *rsmA4* mutants. (C) *Pst*DC3000 and the *rsmA2/rsmA3*, *rsmA2/rsmA4*, *rsmA3/rsmA4* and *rsmA2/rsmA3/rsmA4* mutants. All the strains were grown in KB at 28 °C. Overnight cultures of *Pst*DC3000, mutants and overexpression strains as well as complementation strains were harvested and resuspended to OD_600_ = 0.01 in fresh KB medium. Bacterial strains were grown at 28 °C, and aliquots of the culture were taken every 2 h for 24 h. Bacterial growth for each strain was determined by measuring OD_600_. The experiments were performed in triplicate and repeated three times and similar results were obtained. Vertical bars represent standard deviations.

**Fig. S7** HR assay on tobacco leaves. *Pst*DC3000, the *rsmA* overexpression and *rsmA* mutant strains were infiltrated into 8‐week‐old tobacco leaves. PBS was used as negative control. Photographs were taken 24 h post‐infiltration. The experiment was repeated three times and similar results were obtained. Overnight cultures of bacterial strains were harvested by centrifugation, resuspended in 1/2× PBS and adjusted to OD_600_ = 0.1. Bacterial suspension was infiltrated into tobacco leaves (*Nicotiana tabacum*) by needleless syringe. Infiltrated plants were kept in a humid growth chamber and HR symptoms were recorded at 24 h post‐infiltration. The experiment was repeated three times.

**Fig. S8** Protease activity by *P.* *syringae* pv. *tomato* DC3000, *rsmA* overexpression, *rsmA* mutants and complementation strains. (A) *Pst*DC3000(pUCP18), *Pst*DC3000(pRsmA2), *Pst*DC3000(pRsmA3) and *P* *st*DC3000(pRsmA4) overexpression strains. (B) *Pst*DC3000 and the *rsmA2*, *rsmA3*, *rsmA4*, *rsmA2/rsmA3*, *rsmA2/rsmA4*, *rsmA3/rsmA4* and *rsmA2/rsmA3/rsmA4* mutants. (C) *Pst*DC3000, the *rsmA2/rsmA3* and *rsmA2/rsmA3/rsmA4* mutants and their complementation strains. Protease activity was measured at room temperature using NYG agar plates containing 0.75% skimmed milk where halo zones are indicative of protease activities. Pictures were taken at 72 h post‐incubation. The experiment was repeated three times and similar results were obtained.

**Fig. S9** Pyoverdine production by *P.* *syringae* pv. *tomato* DC3000, *rsmA* overexpression, *rsmA* mutants and complementation strains. (A) *Pst*DC3000(pUCP18), *Pst*DC3000 (pRsmA2), *Pst*DC3000(pRsmA3) and *Pst*DC3000(pRsmA4) overexpression strains. (B) *Pst*DC3000 and the *rsmA2*, *rsmA3*, *rsmA4*, *rsmA2/rsmA3*, *rsmA2/rsmA4*, *rsmA3/rsmA4* and *rsmA2/rsmA3/rsmA4* mutants. (C) *Pst*DC3000, the *rsmA2/rsmA3* and *rsmA2/rsmA3/rsmA4* mutants and their complementation strains. Pyoverdine production was visualized on MG plates under the UV light, where intensities of fluorescence were indicative of pyoverdine production. All strains were grown on MG plates at 28 °C. Pictures were taken at 48 h post‐incubation. The experiment was repeated three times and similar results were obtained.

**Fig. S10** Motility of *P.* *syringae* pv. *tomato* DC3000, *rsmA* overexpression, *rsmA* mutants and complementation strains. (A) *Pst*DC3000, *Pst*DC3000(pUCP18), *Pst*DC3000(pRsmA2), *Pst*DC3000(pRsmA3), *Pst*DC3000(pRsmA4) overexpression strains and the *rsmA2*, *rsmA3* and *rsmA4* single mutant strains. (B) The *rsmA2/rsmA3*, *rsmA2/rsmA4*, *rsmA3/rsmA4* and *rsmA2/rsmA3/rsmA4* mutants. (C) *Pst*DC3000, the *rsmA2/rsmA3* and *rsmA2/rsmA3/rsmA4* mutants and complementation strains. All strains were grown on 0.3% KB agar plates at room temperature. Pictures were taken at 24 h and 48 h post‐inoculation. The experiment was repeated three times and similar results were obtained.

**Fig. S11** Diameter of movement circle of *P.* *syringae* pv. *tomato* DC3000, *rsmA* overexpression, *rsmA* mutants and complementation strains. (A) *Pst*DC3000, *Pst*DC3000(pUCP18), *Pst*DC3000(pRsmA2), *Pst*DC3000(pRsmA3) and *Pst*DC3000(pRsmA4) overexpression strains and the *rsmA2*, *rsmA3* and *rsmA4* single mutant strains. (B) *Pst*DC3000 and the *rsmA2/rsmA3*, *rsmA2/rsmA4*, *rsmA3/rsmA4* and *rsmA2/rsmA3/rsmA4* mutants. (C) *Pst*DC3000, the *rsmA2/rsmA3/rsmA4* mutant and its complementation strains. (D) *Pst*DC3000, the *rsmA2/rsmA3* mutant and its complementation strains. All strains were grown on 0.3% KB agar plates at room temperature. Diameters of the movement circles were measured at 24 h and 48 h post‐incubation. Vertical bars represent standard deviations. One‐way ANOVA and the Student–Newman–Keuls test (*P* = 0.05) were used to analyse motility diameter data. Bars marked with the same letter are not significantly different (*P* < 0.05). The experiment was repeated three times and similar results were obtained.

**Fig. S12** Duplicated SDS‐PAGE gel stained using Coomassie Blue as loading control. (A) 1, marker; 2, DC3000(pRsmA2‐His); 3, ∆*rsmA2*(pRsmA2‐His); 4, ∆*rsmA3*(pRsmA2‐His); 5, ∆*rsmA4*(pRsmA2‐His); 6, DC3000(pRsmA3‐His); 7, ∆*rsmA2*(pRsmA3‐His); 8, ∆*rsmA3(*pRsmA3‐His); 9, ∆*rsmA4*(pRsmA3‐His); 10, DC3000(pRsmA4‐His); 11, ∆*rsmA2*(pRsmA4‐His); 12, ∆*rsmA3*(pRsmA4‐His); 13, ∆*rsmA4*(pRsmA4‐His). (B) 1, marker; 2, DC3000(pRsmA2‐His); 3, DC3000(pRsmA3‐His); 4, DC3000(pRsmA4‐His); 5, ∆*rsmA2/A3*(pRsmA2‐His); 6, ∆*rsmA2/A3*(pRsmA3‐His); 7, ∆*rsmA2/A3*(pRsmA4‐His); 8, ∆*rsmA2/A3/A4*(pRsmA2‐His); 9, ∆*rsmA2/A3/A4*(pRsmA3‐His); 10, ∆*rsmA2/A3/A4*(pRsmA4‐His).

**Table S1** Comparison of deduced amino acids of different RsmA/CsrA proteins in *E. amylovora* and three *Pseudomonas* strains.

**Table S2** Primers used in this study.
