# Supplementary figures and images for: Homologues of the RNA binding protein RsmA in Pseudomonas syringae pv. tomato DC3000 exhibit distinct binding affinities with non‐coding small RNAs and have distinct roles in virulence
Source: Mol Plant Pathol. 2019 Jun 20;20(9):1217–36. doi: 10.1111/mpp.12823 (PMC6715622; doi:10.1111/mpp.12823)

**A**

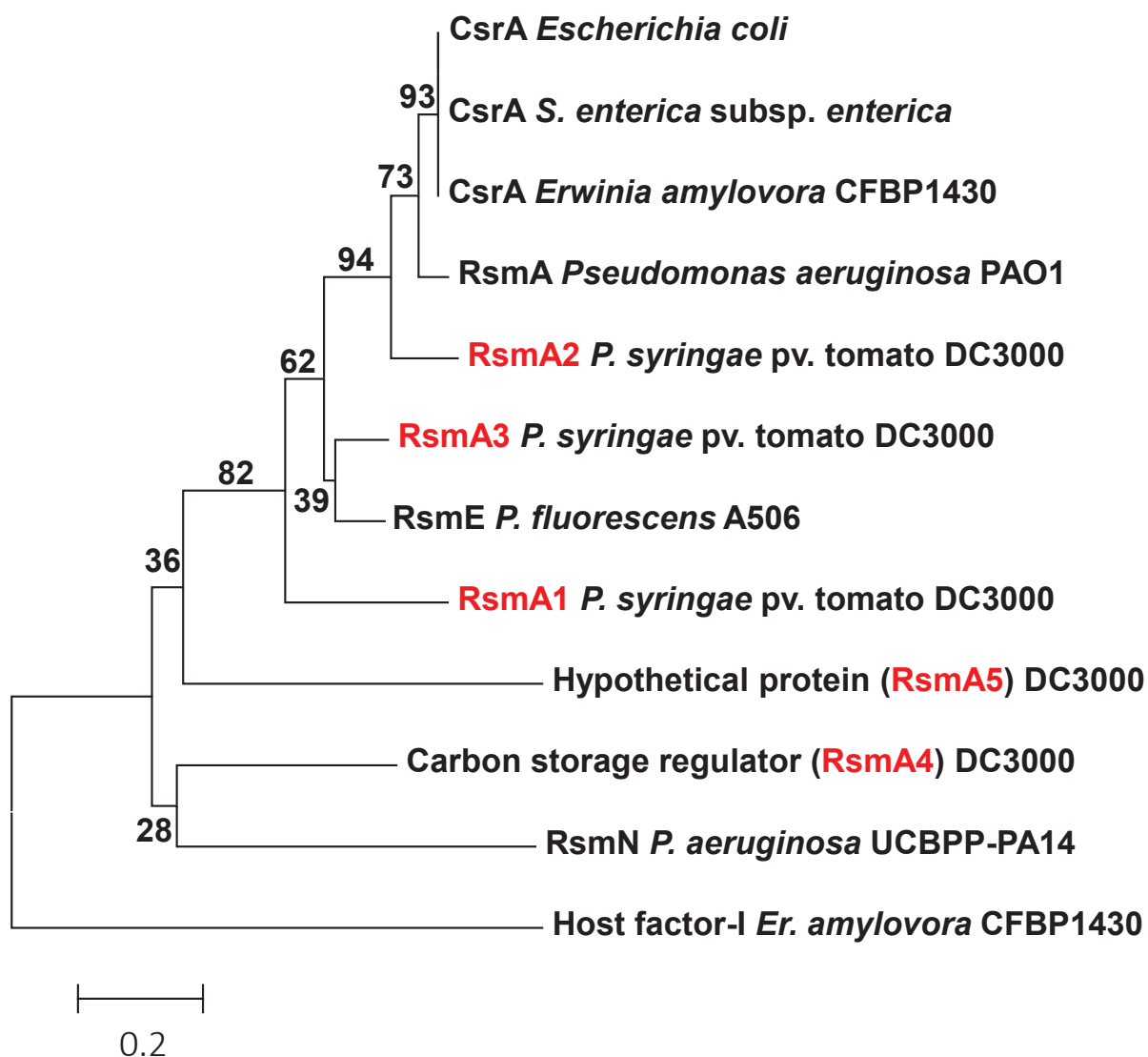

**B**

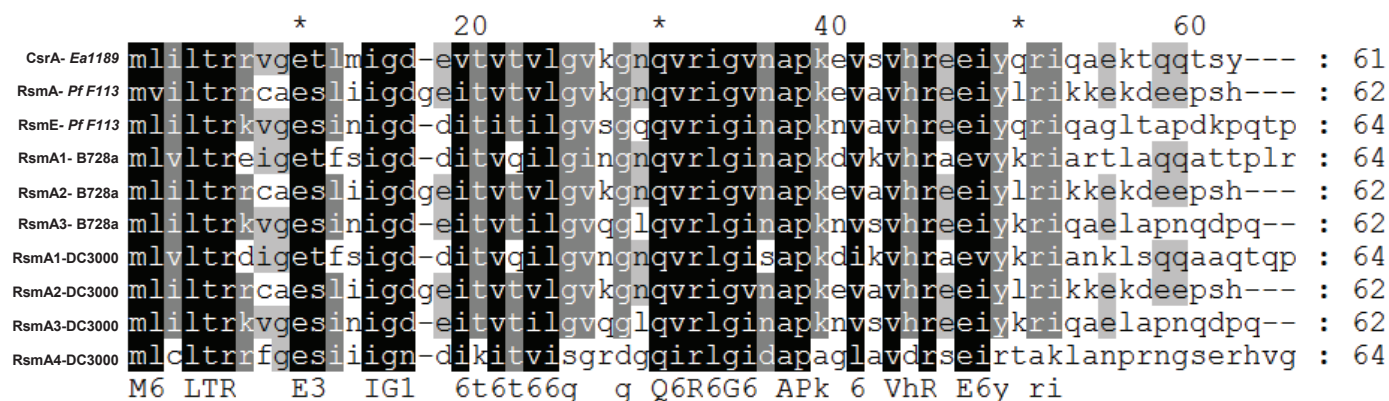

**Fig. S1**

Supplement: Supplementary file 1 — Fig. S1 (A) Phylogenetic tree of RsmA/CsrA proteins from E. coli, S. enterica, E. amylovora and Pseudomonas strains. (B) Alignment of deduced amino acids of different RsmA/CsrA proteins in E. amylovora and three Pseudomonas strains. The deduced amino acid sequences of RsmA/CsrA proteins were aligned and analysed by GeneDoc software (Nicholas et al., 1997). Phylogenetic tree of RsmA/CsrA proteins was made by MEGA5 (Tamura et al., 2001). The GenBank accession numbers are E. coli CsrA: BAA16558; S. enterica subsp. enterica CsrA: NP_461747; E. amylovora CFBP1430 CsrA: CBA19758; Host factor‐I: CBA23141; P. aeruginosa PAO1 RsmA: AAG04294; P. aeruginosa UCBPP‐PA14 RsmN: BAK92751; P. fluorescens F113 RsmA: ABW16952; RsmE: ABW16953; P. fluorescens A506 RsmE: AFJ58988; P. syringae pv. syringae B728a RsmA1: YP_236820; RsmA2: YP_236624; RsmA3: YP_236409; P. syringae pv. tomato DC3000 RsmA1: AAO55149; RsmA2: AAO55363; RsmA3: AAO57040; RsmA4: AAO57404; RsmA5: YP_003355050. [file MPP-20-1217-s001.pdf]

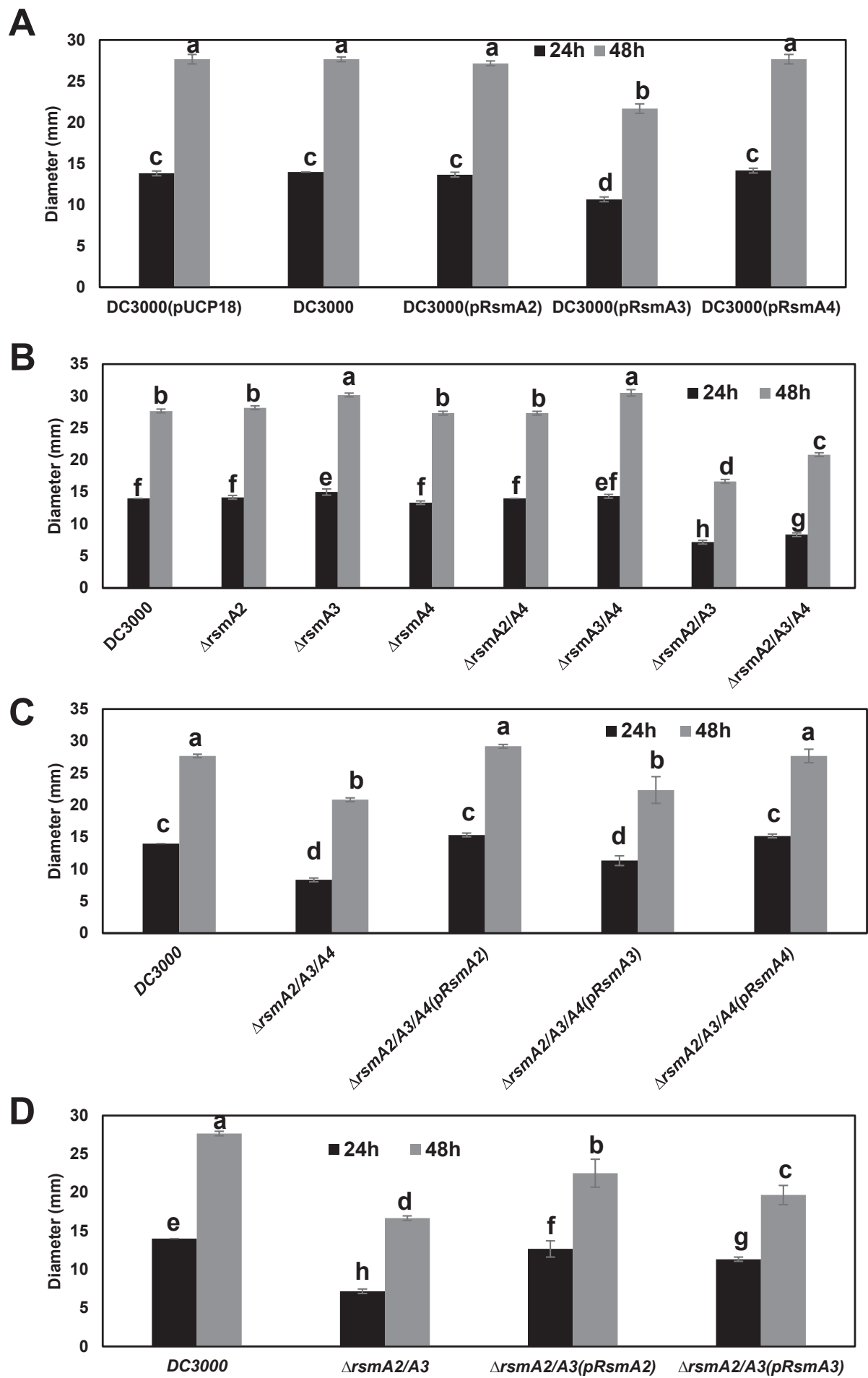

**Fig. S11**

Supplement: Supplementary file 11 — Fig. S11 Diameter of movement circle of P. syringae pv. tomato DC3000, rsmA overexpression, rsmA mutants and complementation strains. (A) PstDC3000, PstDC3000(pUCP18), PstDC3000(pRsmA2), PstDC3000(pRsmA3) and PstDC3000(pRsmA4) overexpression strains and the rsmA2, rsmA3 and rsmA4 single mutant strains. (B) PstDC3000 and the rsmA2/rsmA3, rsmA2/rsmA4, rsmA3/rsmA4 and rsmA2/rsmA3/rsmA4 mutants. (C) PstDC3000, the rsmA2/rsmA3/rsmA4 mutant and its complementation strains. (D) PstDC3000, the rsmA2/rsmA3 mutant and its complementation strains. All strains were grown on 0.3% KB agar plates at room temperature. Diameters of the movement circles were measured at 24 h and 48 h post‐incubation. Vertical bars represent standard deviations. One‐way ANOVA and the Student–Newman–Keuls test (P = 0.05) were used to analyse motility diameter data. Bars marked with the same letter are not significantly different (P < 0.05). The experiment was repeated three times and similar results were obtained. [file MPP-20-1217-s011.pdf]
