# Supplementary material for: Homologues of the RNA binding protein RsmA in Pseudomonas syringae pv. tomato DC3000 exhibit distinct binding affinities with non‐coding small RNAs and have distinct roles in virulence
Source: Mol Plant Pathol. 2019 Jun 20;20(9):1217–36. doi: 10.1111/mpp.12823 (PMC6715622; doi:10.1111/mpp.12823)
Supplement: Supplementary file 13 — Table S1 Comparison of deduced amino acids of different RsmA/CsrA proteins in E. amylovora and three Pseudomonas strains. [file MPP-20-1217-s013.docx]

**Table S1.** Comparison of deduced amino acids of different RsmA/CsrA proteins in *E. amylovora* and three *Pseudomonas* strains

|  | **CsrA-**  ***E. amylovora*** | **RsmA*-***  ***P. fluorescens***  ***F113*** | **RsmE*-***  ***P. fluorescens F113*** | **RsmA1-**  **B728a** | **RsmA2-**  **B728a** | **RsmA3-**  **B728a** | **RsmA1-**  **DC3000** | **RsmA2-**  **DC3000** | **RsmA3-**  **DC3000** | **RsmA4-**  **DC3000** |
| --- | --- | --- | --- | --- | --- | --- | --- | --- | --- | --- |
| Query coverage percentage | **100** | **100** | **87** | **98** | **100** | **89** | **98** | **100** | **89** | **77** |
| **CsrA-**  ***E. amylovora*** | **Identity 100%** | **74** | **64** | **54** | **75** | **67** | **53** | **75** | **67** | **34** |
|  | **Similarity 100%** | **88** | **78** | **75** | **88** | **82** | **78** | **88** | **82** | **59** |
| **RsmA*-***  ***P. fluorescens***  ***F113*** |  | **100** | **56** | **47** | **98** | **60** | **46** | **98** | **60** | **35** |
|  |  | **100** | **69** | **70** | **100** | **74** | **72** | **100** | **74** | **53** |
| **RsmE*-***  ***P. fluorescens***  ***F113*** |  |  | **100** | **54** | **58** | **76** | **54** | **58** | **76** | **42** |
|  |  |  | **100** | **73** | **69** | **84** | **75** | **69** | **84** | **57** |
| **RsmA1-**  **B728a** |  |  |  | **100** | **49** | **59** | **81** | **49** | **59** | **32** |
|  |  |  |  | **100** | **70** | **73** | **89** | **70** | **73** | **51** |
| **RsmA2-**  **B728a** |  |  |  |  | **100** | **61** | **47** | **100** | **61** | **36** |
|  |  |  |  |  | **100** | **74** | **72** | **100** | **74** | **53** |
| **RsmA3-**  **B728a** |  |  |  |  |  | **100** | **56** | **61** | **100** | **39** |
|  |  |  |  |  |  | **100** | **73** | **74** | **100** | **59** |
| **RsmA1-DC3000** |  |  |  |  |  |  | **100** | **47** | **56** | **32** |
|  |  |  |  |  |  |  | **100** | **72** | **73** | **50** |
| **RsmA2-DC3000** |  |  |  |  |  |  |  | **100** | **61** | **36** |
|  |  |  |  |  |  |  |  | **100** | **74** | **53** |
| **RsmA3-DC3000** |  |  |  |  |  |  |  |  | **100** | **39** |
|  |  |  |  |  |  |  |  |  | **100** | **59** |
| **RsmA4-DC3000** |  |  |  |  |  |  |  |  |  | **100** |
|  |  |  |  |  |  |  |  |  |  | **100** |

The gene bank accession numbers are: *E. amylovora* CFBP1430 CsrA: CBA19758; *P. fluorescens* RsmA: ABW16952; *P. fluorescens* RsmE: ABW16953; *P. syringae* pv. *syringae* B728a RsmA1: YP_236820; RsmA2: YP_236624; RsmA3: YP_236409; *P. syringae* pv. *tomato* DC3000 RsmA1: AAO55149; RsmA2: AAO55363; RsmA3: AAO57040; RsmA4: AAO57404.
