# Supplementary material for: Homologues of the RNA binding protein RsmA in Pseudomonas syringae pv. tomato DC3000 exhibit distinct binding affinities with non‐coding small RNAs and have distinct roles in virulence
Source: Mol Plant Pathol. 2019 Jun 20;20(9):1217–36. doi: 10.1111/mpp.12823 (PMC6715622; doi:10.1111/mpp.12823)
Supplement: Supplementary file 14 — Table S2 Primers used in this study. [file MPP-20-1217-s014.docx]

**Table S2.** Primers used in this study

| **Primer** | **Sequences (5′–3′)** |
| --- | --- |
| **Mutagenesis** | |
| rsmA1 F1  rsmA1 R1  rsmA1 F2  rsmA1 R2  rsmA2 F1 | GCCGATTACATCTTCACGCA  GAAGCAGCTCCAGCCTACACCTGCCTCTTCGTTACGCAAA  GGTCGACGGATCCCCGGAATAAACTCTTTGGGCACATCGT  TGCCAGAGTTCACCGAGAG  TGACGAGTGCCAGATCTACA |
| rsmA2 R1 | GAAGCAGCTCCAGCCTACACACCGACGAGTCAGAATCAGC |
| rsmA2 F2 | GGTCGACGGATCCCCGGAATGAAGGACGAAGAACCAAGCC |
| rsmA2 R2 | CGAGGACGCATCAGGAAAGA |
| rsmA3 F1 | AGCGGTGTGGGTGATGTC |
| rsmA3 R1 | GAAGCAGCTCCAGCCTACACCTCTTGAAAACGCCTCTCTCA |
| rsmA3 F2 | GGTCGACGGATCCCCGGAATCCAAACCAGGACCCACAATA |
| rsmA3 R2 | ACGACGACTATTCAAAGCGC |
| rsmA4 F1 | GTGCCTCTGATTTACGCCG |
| rsmA4 R1 | GAAGCAGCTCCAGCCTACACCCAAAGCAGTTAATGAATCAGCG |
| rsmA4 F2 | GGTCGACGGATCCCCGGAATCCATGTCGGCTGATAATCGA |
| rsmA4 R2 | CCCGCAGAATCTCGTCCTC |
| FRT-Km-FRT F | GTGTAGGCTGGAGCTGCTTC |
| FRT-Km-FRT R | ATTCCGGGGATCCGTCGACC |
| **Complementation and cloning, western blot** | |
| RsmA1com-F (EcoRI) | AGTC*GAATTC*CCCACCGGTCAGAAGCCATC |
| RsmA1com-R (XbaI) | AGTC*TCTAGA*GACACCCTCACCCGCCTGAT |
| RsmA2com-F (EcoRI) | AGTC*GAATTC*GGTGTGCCAGACACCCCCGG |
| RsmA2com-R (XbaI)  RsmA2-His6-R (XbaI) | AGTC*TCTAGA*TTCAATAACTTACCGATCAA  CCG*TCTAGA*TCA**GTGGTGGTGGTGGTGGTG**ATGGCTTGGTTCTTCGTCCT |
| RsmA3com-F (EcoRI) | AGTCGAATTCTGGTCGGCCTGATCTTCATC |
| RsmA3com-R (XbaI)  RsmA3-His6-R (XbaI) | AGTCTCTAGAGGCTCGCGCACAAGGCCTCA  CCGTCTAGATCA**GTGGTGGTGGTGGTGGTG**TTGTGGGTCCTGGTTTGGAG |
| RsmA4com-F (EcoRI) | AGTCGAATTCTCGAAGGGTTATCCCCTGTC |
| RsmA4com-R (XbaI)  RsmA4-His6-R (XbaI) | AGTCTCTAGACTTGATCTATCAGTCGGCGC  CCG*TCTAGA*TCA**GTGGTGGTGGTGGTGGTG**GCCGACATGGCGCTCACTCC |
| CsrAcom-F (KpnI) | AGTA*GGTACC*AAAAGTAGGCGGTAAAGG |
| CsrAcom-R (XbaI)  **Small RNA expression**  RsmX1-F  RsmX1-R  RsmX5-F  RsmX5-R  RsmY-F | AGTA*TCTAGA*TGCCTAAACCAGCTTAATGGA  ATCAACTGCAGCGCAGGAAGC  AAAAAAACCCGCCGAAGCGGG  ATCAACTGGTGAACAGGAGTT  AAAAAACCCGCCGAAGCGGGT  ATGGACGTAGCGCAGGAAGCG |
| RsmY-R  RsmZ-F  RsmZ-R  **Protein expression**  pET42b-RsmA1-His6-F (Ndel)  pET42b-RsmA1-His6-R (Xhol)  pET42b-RsmA2-His6-F (Ndel)  pET42b-RsmA2-His6-R (Xhol)  pET42b-RsmA3-His6-F (Ndel)  pET42b-RsmA3-His6-R (Xhol)  pET42b-RsmA4-His6-F (Ndel)  pET42b-RsmA4-His6-R (Xhol) | AAAGAAAACCCCGCCTAAGCG  TGTGCCAACGGACAGGCACAG  AAAAAAAGGGGCGGTATGACC  GGGAATTC*CATATG*CTGGTATTGACGCGGGA  CCG*CTCGAG*TCA**GTGGTGGTGGTGGTGGTG**TGGCTGCGTCTGCGCGGCCT  GGGAATTC*CATATG*CTGATTCTGACTCGTCG  CCG*CTCGAGT*CA**GTGGTGGTGGTGGTGGTG**ATGGCTTGGTTCTTCGTCCT  GGGAATTC*CATATG*TTGATACTCACTCGCAA  CCG*CTCGAG*TCA**GTGGTGGTGGTGGTGGTG**TTGTGGGTCCTGGTTTGGAG  GGGAATTC*CATATG*CTGTGTCTAACCCGTCG  CCG*CTCGAG*TCA**GTGGTGGTGGTGGTGGTG**GCCGACATGGCGCTCACTCC |
| **qRT-PCR** | |
| rpoD-F | GAGAACGTCGGAGAGACGAC |
| rpoD-R | GAAGGCATCCGTGAAGTGAT |
| avrE-F | CTGCCACGCCGCCCACCGCG |
| avrE-R | GGGGCTGCCTCCGGGTGTAT |
| hrpL-F | CTCCAGTGCGTGTTTCTTGA |
| hrpL-R | GATGCCCCTCTACCTGATGA |
| corR-F | GGCCCTGATGTTGGAAATAA |
| corR-R | GCAAATCGAGGAGGATGAGA |
| cfl-F | GAGCAGTATCTGCCCTGGTC |
| cfl-R | GGTGCCGGACGTATAGAAAA |
| algQ-F | GGCGACACACCCGAGGCATT |
| algQ-R | GTCAACGCAAACTTGGTGAT |
| algK-F | TTCAGGTGAATACCCGCGAC |
| algK-R | AGCGTGCCCGACTCACCGTT |
| rsmA1-F  rsmA1-R  rsmA2-F | TGACATCACCGTGCAGATTC  GCCCGGTGAACCTTGATGT  TGAAATCACCGTAACCGTGC |
| rsmA2-R | AATGGCTTGGTTCTTCGTCC |
| rsmA3-F | GAGATCACCGTCACGATTCT |
| rsmA3-R | GGAATTATTGTGGGTCCTGGT |
| rsmA4-F | CTGTGTCTAACCCGTCGCTT |
| rsmA4-R | ATGGCGCTCACTCCCGTT |

Underlined italics: restriction sites; underlined bold: His6-tags.
